# From the flagellum attachment zone to the tripartite attachment complex – widespread cytoskeletal engagement of *T. brucei* KMP11

**DOI:** 10.1242/jcs.264898

**Published:** 2026-08-20

**Authors:** Salome Aeschlimann, Clirim Jetishi, Caroline E. Dewar, Philip Stettler, Markus Gerber, Silke Oeljeklaus, Bettina Warscheid, Torsten Ochsenreiter, André Schneider

**Affiliations:** ^1^ Department of Chemistry, Biochemistry and Pharmaceutical Sciences, University of Bern, 3012 Bern, Switzerland; ^2^ Institute of Cell Biology, University of Bern, 3012 Bern, Switzerland; ^3^ Division of Biomedical and Life Sciences, Faculty of Health and Medicine (FHM), Lancaster University, Lancaster LA1 4YW, UK; ^4^ Faculty of Chemistry and Pharmacy, Biochemistry II, Theodor Boveri-Institute, Biocenter, University of Würzburg, 97074 Würzburg, Germany

**Keywords:** *T. brucei*, Cytoskeleton, U-ExM, mtDNA inheritance, Tripartite attachment complex

## Abstract

KMP11 is a conserved kinetoplastid protein yet its precise localization and function remained unclear. Using immunofluorescence, ultrastructure expansion microscopy (U-ExM), and proteomics, we comprehensively analyzed KMP11 in procyclic *Trypanosoma brucei*. Besides confirming localization to the flagellum attachment zone (FAZ), we identify previously unknown localizations at the paraflagellar rod (PFR), flagellar connector (FC), microtubule quartet (MtQ) and flagellar pocket collar/hook complex (FPC/HC) region. We further show that the previously described basal body localization corresponds to the tripartite attachment complex (TAC). Proteomic analyses identify multiple cytoskeletal interactors, including the TAC component p197. Immunoprecipitation and U-ExM analyses demonstrate that KMP11 associates with the central α-helical domain of p197 from both *T. brucei* and *Trypanosoma cruzi*. Furthermore, KMP11 interactors overall display significantly elevated α-helical content. Together, our findings establish KMP11 as a component of a broad cytoskeletal interaction network in trypanosomes and we speculate that KMP11 preferentially associates with α-helical proteins.

## Teaser

**Summary:** *In Trypanosoma brucei*, KMP11 is an essential protein that binds α-helical domains, such as the TAC protein p197, and likely functions as a crosslinker connecting multiple such cytoskeletal structures.

## INTRODUCTION

The kinetoplastid membrane protein 11 (KMP11) is an abundant kinetoplastid-specific protein of 11 kDa originally identified as a T-cell stimulatory surface antigen in the parasitic protozoan *Leishmania donovani* (Jardim et al., 1995a,b). KMP11 is highly immunogenic and has been proposed as a vaccine candidate against leishmaniasis (de Mendonca et al., 2015). In *Leishmania braziliensis*, KMP11 is expressed in promastigotes and amastigotes, and localizes to the cell surface, flagellar pocket and intracellular vesicles (Matos et al., 2010). Another study has found that in promastigote *Leishmania amazonensis*, the protein predominantly localizes to the flagellum and the basal body (BB) (Berberich et al., 1998). Finally, a recent study in *L. donovani* has suggested that KMP11 contains cholesterol-interaction motifs (known as CRAC and CARC motifs), which might mediate cholesterol transfer from macrophages to the parasite membrane and facilitate parasite invasion. Accordingly, KMP11-knockout cells are deficient for macrophage invasion but also show an eightfold reduction in cell volume and severely impaired growth (Sannigrahi et al., 2024).

Trypanosomal and leishmanial KMP11 are homologs, but their intracellular localizations appear surprisingly different. Immunofluorescence (IF) analysis in procyclic (PCF) and bloodstream form (BSF) *Trypanosoma brucei* shows that tagged KMP11 is localized to the BB and the flagellum (Li and Wang, 2008; Sunter et al., 2023). Moreover, this flagellar staining includes the flagellum attachment zone (FAZ), which connects the flagellum to the cell body and guides cytokinesis (de Liz et al., 2023; Sunter and Gull, 2016). Finally, the tagged KMP11 weakly stains the nucleus (Zhou et al., 2015). The flagellar and BB localization of *T. brucei* KMP11 are in line with the reported localization of the protein in *L. amazonensis* (Berberich et al., 1998). However, unlike its counterpart in *Leishmania* species (Jardim et al., 1995b; Matos et al., 2010), there is no evidence that the *T. brucei* KMP11 localizes to intracellular vesicles or the surface of the parasite.

There are also differences between trypanosomal and leishmanial KMP11 depletion phenotypes. Whereas *L. donovani* cells depleted of KMP11 remain viable (Sannigrahi et al., 2024), tetracycline-inducible RNAi targeting *T. brucei* KMP11 in either PCFs or BSFs causes a fast growth arrest and subsequent cell death, beginning within 24 h of RNAi induction, demonstrating that *T. brucei* KMP11 is a highly essential protein (Li and Wang, 2008). Induced KMP11 RNAi *T. brucei* cells show impaired BB segregation and cytokinesis with subsequent accumulation of multinucleated cells. Moreover, in PCFs, the RNAi also disrupts new FAZ formation and causes detachment of the new flagellum from the cell body (de Liz et al., 2023). In agreement with this finding, KMP11 has been reported to interact with the FAZ component FAZ2 (Zhou et al., 2015). Flagellum detachment is not observed in BSFs (Li and Wang, 2008).

The secondary structure of KMP11 consists mainly of α-helices, large parts of which are of amphiphilic nature. For the 3D structure of the protein, two very different conformations have been proposed. In aqueous solution, AlphaFold predicts a high confidence (pLDDT=79.81) helix-turn-helix fold where the helices are ∼30 and ∼47 amino acids (aa) in length (https://alphafold.ebi.ac.uk/entry/AF-Q9U6Z1-F1; last accessed Jan 01, 2026) (Jumper et al., 2021). This prediction is confirmed by a crystal structure showing an antiparallel homodimer of two single KMP11 chains (https://www.rcsb.org/3d-view/7F0K, last accessed Jan 01, 2026). In contrast, NMR analysis has shown that, in the presence of dodecylphosphocholine (DPC)-micelles, mimicking a membrane environment, a single KMP11 molecule adopts a stable four helix bundle fold (Lim et al., 2017).

In this short communication, we report new localizations of *T. brucei* KMP11, including the tripartite attachment complex (TAC), which we analyzed in greater detail.

## RESULTS AND DISCUSSION

### Expansion microscopy reveals new localizations of KMP11

**Fig. 1.**
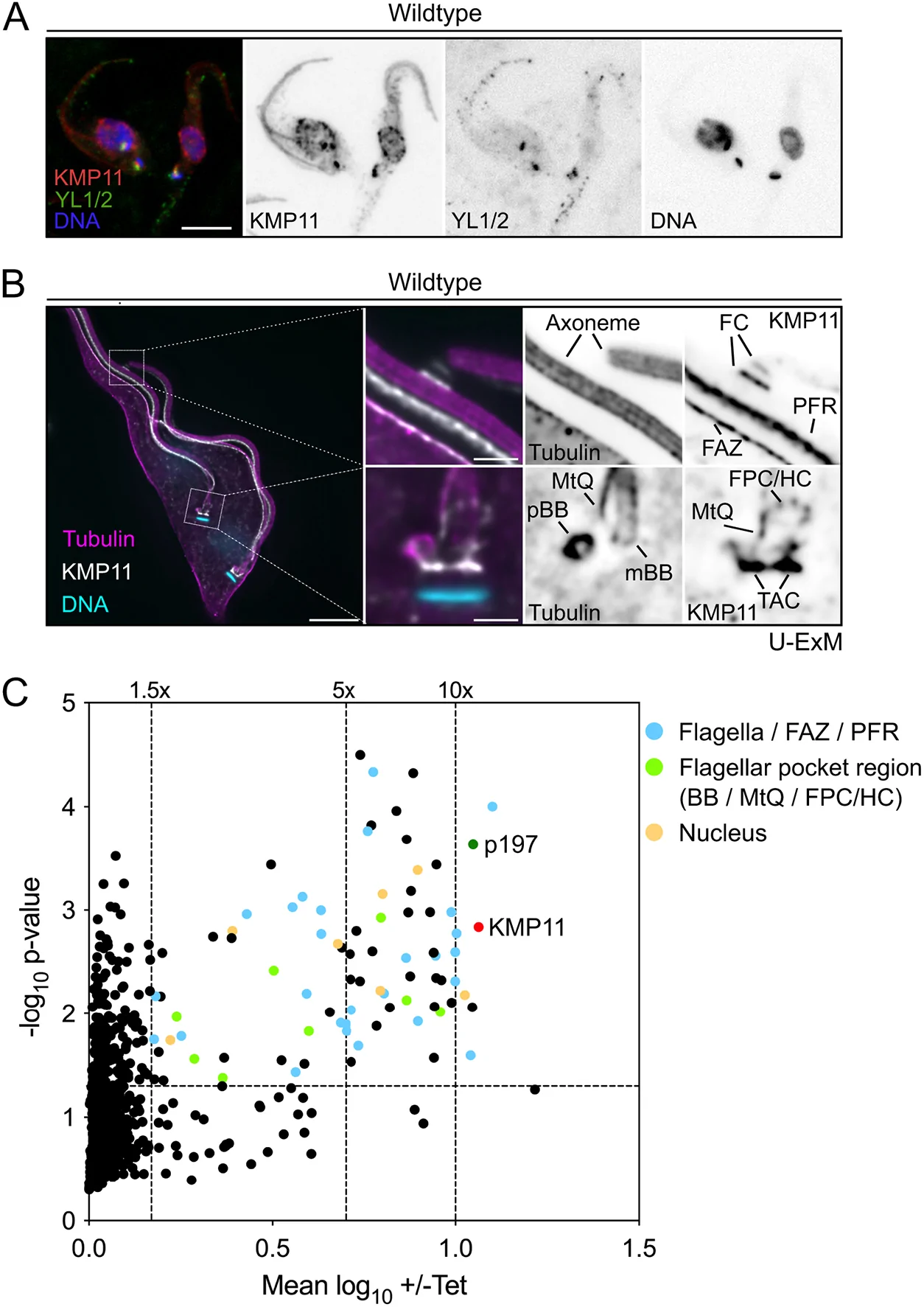
**The interaction network of KMP11.** (A) Immunofluorescence analysis of wild-type PCF cells stained for KMP11 (red), YL1/2 as a BB marker (green) and with DAPI for the DNA (blue). Scale bar: 5 μm. (B) U-ExM of wild-type PCF cells stained for KMP11 (white), 6-11B-1 antibody for α-tubulin (magenta) and DAPI for the DNA (blue). Visible structures are indicated. Scale bar: 10 μm (left), 2 μm (enlargement). FC, flagella connector; PFR, paraflagellar rod; FAZ, flagellum attachment zone; HC, hook complex; FPC, flagellar pocket collar; MtQ, microtubule quartet; TAC, tripartite attachment complex. For A, B, images representative of hundreds of cells from at least three repeats. (C) Visualization of proteins identified in SILAC-based quantitative MS analyses of KMP11–Myc immunoprecipitations from PCF cells (*n*=3). Enrichment factors (1.5×, 5× and 10×) are indicated by vertical dotted lines. A *P-*value of 0.05 is indicated by the horizontal dotted line. The following proteins or groups of proteins (≥1.5-fold enriched, *P*<0.05) are highlighted: KMP11 (red), p197 (dark green), flagellar proteins including FAZ and PFR (blue), flagellar pocket region proteins (light green), and nuclear proteins (yellow). For numerical data, see Tables S1 and S2. In all U-ExM panels, the scale bars refer to the images as acquired (i.e. to the expanded specimen).

To get a more complete picture of KMP11 localization in PCF *T. brucei*, we performed an IF analysis using a polyclonal antibody recognizing the untagged protein (Fig. 1A). Cells were co-stained with the monoclonal YL1/2 antibody, which recognizes tyrosinated α-tubulin and, in *T. brucei*, also the BB protein TbRP2 (Stephan et al., 2007; Andre et al., 2014). The results showed that KMP11 was present in the BB region, along the old and the new flagellum and at the nuclear periphery, which in agreement with previous reports (Li and Wang, 2008).

Next, we did the same analysis using ultrastructure expansion microscopy (U-ExM), which allows nanoscale spatial resolution. The only difference was that microtubules were stained using the monoclonal 6-11B-1 antibody, which recognizes acetylated α-tubulin (Fig. 1B). This revealed flagellar staining of KMP11 in two distinct localizations. One signal started immediately outside the flagellar pocket and continued along the axoneme. However, it did not overlap with it, indicating a localization to the paraflagellar rod (PFR) (Ralston and Hill, 2008; Lacomble et al., 2009; Kohl et al., 1999). The second signal stained the region of the cell body adjacent to the flagellum corresponding to the cytosolic membrane-associated FAZ filament (de Liz et al., 2023; Sunter and Gull, 2016). FAZ filament localization is also supported by RNAi experiments targeting KMP11 where after 18 h of RNAi induction the new flagellum detached from the cell body in many cells (Fig. S1A,B). Moreover, KMP11 was also present in the flagellar connector (FC), a mobile transmembrane structure tethering the tip of the new flagellum to the side of the old flagellum (Moreira-Leite et al., 2001). Analysis of the morphology and dimensions of the FC-associated IF signals indicated that different amounts of KMP11 were present in two subdomains of the FC – the old and the new flagellum layer (Hoog et al., 2016; Varga et al., 2017).

We also reinvestigated the previously reported BB localization of KMP11. Although the 6-11B-1 α-tubulin antibody stained the axoneme and the BB, KMP11 localized to the region between the BB and trypanosomal mitochondrial genome, the kinetoplast DNA (kDNA) (Fig. 1B). This places KMP11 at the TAC, which links kDNA to the BB and mediates the segregation of the replicated single nucleoid mitochondrial genomes (Fig. 1B; Aeschlimann et al., 2023; Schneider and Ochsenreiter, 2018). In addition, KMP11 localization was detected in the kDNA-flagellar pocket region encompassing the microtubule quartet (MtQ) and the flagellar pocket collar (FPC)/hook complex region (HC) (Fig. 1B; Wheeler et al., 2019). Note, although little or no nuclear staining was found for KMP11 in our U-ExM, a nuclear localization is seen using conventional microscopy (Zhou et al., 2015) (Fig. 1A).

To verify antibody specificity, total cell extracts prepared following 18 h of KMP11 RNAi induction were analyzed by western blotting alongside uninduced controls, revealing specific depletion of an 11 kDa band (Fig. S1C). Antibody specificity was further corroborated by U-ExM analysis of ectopically expressed epitope-tagged KMP11, which recapitulated the localization pattern of the endogenous protein at the FAZ, PFR, TAC and FC (Fig. S2). Interestingly, western blot analysis showed that the protein levels of ectopically expressed KMP11–Myc were substantially lower than those of endogenous KMP11, rendering the ectopically expressed KMP11–Myc undetectable with the KMP11 antibody (Fig. S3A,B). In addition, ectopic expression of KMP11-HA failed to rescue the growth defect caused by KMP11 RNAi (Fig. S3C,D).

We also identified putative KMP11-interacting proteins by stable isotope labelling by amino acids in cell culture-based immunoprecipitation (SILAC-IP) using Myc-tagged KMP11 as the bait. The resulting eluate contained 50 proteins that were enriched by more than 5-fold (Fig. 1C; Tables S1, S2). A total of 22 (44%) of them have previously been localized to the flagellum (including PFR and FAZ), the flagellar pocket region (including BB, MtQ and the FPC/HC region) or the nucleus. p197, which forms the exclusion zone filaments of the TAC between the BB and the mitochondrial outer membrane (Gheiratmand et al., 2013; Aeschlimann et al., 2022), was among the most enriched proteins with a 11.2-fold enrichment, which was comparable to the enrichment of KMP11 itself (11.6-fold enrichment). FAZ2 and CC2D, previously reported KMP11 interactors, were 12.6- and almost 10-fold enriched, respectively (Zhou et al., 2015). In summary, these results are in line with the U-ExM and IF analysis. It is worth noting that the majority of KMP11 remains insoluble after lysis with detergents such as digitonin, suggesting that very strong interactors might have been missed in the proteomics analysis.

Thus, we have discovered new localizations of KMP11 to the PFR and FC and showed that KMP11 localizes to the TAC rather than the BB itself. Finally, we found that the protein is also present at the MtQ and the FPC/HC region.

### RNAi-mediated depletion of p197 RNAi depletes KMP11

**Fig. 2.**
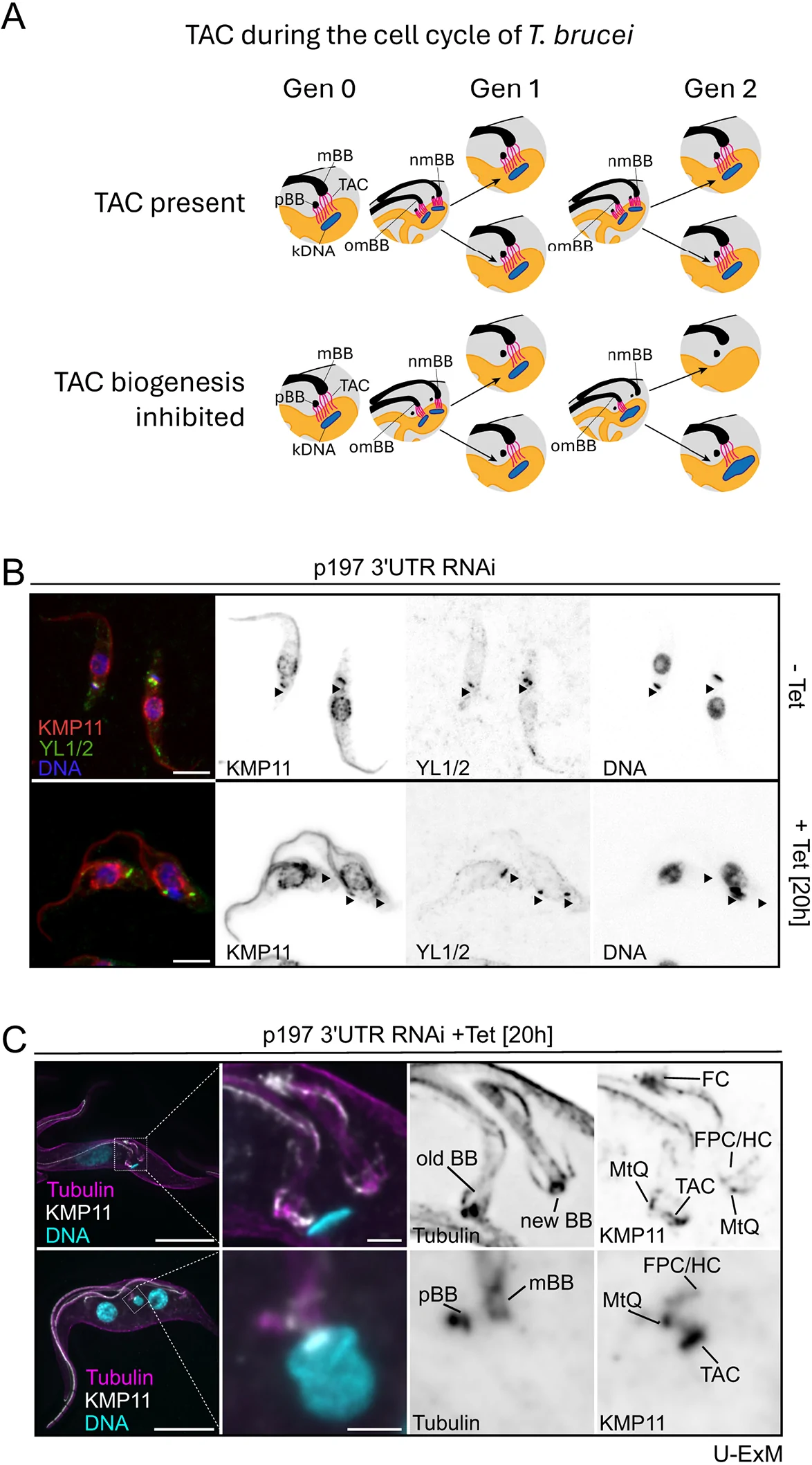
**RNAi-mediated depletion of p197 depletes KMP11 in the TAC region.** (A) Schematic of TAC- biogenesis under normal conditions and p197 RNAi. Abbreviations: TAC, tripartite attachment complex; kDNA, kinetoplast DNA; mBB, mature basal body; pBB, pro-basal body; omBB, old mature basal body; nmBB, new mature basal body. (B) Immunofluorescence analysis of the tetracycline (Tet) inducible p197 3′UTR RNAi PCF cell line uninduced (−Tet) and induced for 20 h (+Tet). Cells have been stained for KMP11 (red), YL1/2 as a BB marker (green) and for the DNA with DAPI (blue). Black arrowheads point towards the region of the BBs. Scale bars: 5 μm. (C) U-ExM of the same cell line as in B induced for 20 h stained for KMP11 (white), 6-11B-1 antibody for α-tubulin (magenta) and DAPI for the DNA (blue). The position of the old, new, mature- (mBB) and pro-BB (pBB) are indicated. Scale bars: 20 μm left, 2 μm (enlargement). Images in B and C are representative of hundreds of cells from at least three repeats. FC, flagellar connector; HC, hook complex; FPC, flagellar pocket collar; MtQ, microtubule quartet; TAC, tripartite attachment complex. In all U-ExM panels, the scale bars refer to the images as acquired (i.e. to the expanded specimen).

The TAC is assembled in a polar way starting from the BB and extending to the kDNA. p197 is the subunit that is most proximal to the BB in the TAC. RNAi-mediated depletion of p197 therefore not only depletes p197, but also all distal TAC subunits (Hoffmann et al., 2018). If KMP11 interacts with the TAC, its localization should depend on p197. Fig. 2A schematically explains the BB–kDNA dynamics during the *T. brucei* cell cycle upon TAC depletion. In brief, under normal conditions, the TAC links the kDNA to the BBs throughout the cell cycle ensuring accurate segregation of the kDNA network into daughter cells (Fig. 2A). In contrast, upon p197 RNAi-mediated inhibition of TAC biogenesis, the newly formed pro-basal bodies fail to assemble TAC structures (Fig. 2A, Gen 0 to Gen 1). As a result, the kDNA remains associated only with the pre-existing BBs during the first generation after depletion (Fig. 2A, Gen 1). Continuous inhibition of TAC biogenesis leads to progressive kDNA missegregation, ultimately resulting in over-replicated kDNA in one daughter cell and complete kDNA loss in the other (Fig. 2A, Gen 2). Conventional IF analysis 20 h after RNAi induction showed the typical TAC depletion phenotype (Fig. 2B) (Schneider and Ochsenreiter, 2018). Besides cells lacking kDNA, a cell population was detected where the old BB was attached to a large over-replicated kDNA, whereas the new BB was devoid of kDNA (Fig. 2B). In these cases, the KMP11 staining was always associated with the old BB attached to the over-replicated kDNA but was much reduced in the region of the new BB that lacks a TAC.

However, KMP11 also localizes to the MtQ which is anchored at the BB. Thus, to determine the localization of KMP11 more precisely, we analyzed p197-depleted cells by U-ExM. As expected, we found numerous cells where the old BB was attached to a large over-replicated kDNA, while the new BB was devoid of kDNA (Fig. 2C). In all these cases, the KMP11 signal was only detected in the TAC region of the old BB, and more precisely, only at the mature BB of the old BB (Fig. 2C). The weak KMP11 signal associated with the new BB that is still detected likely corresponds to the MtQ. As disruption of TAC does not interfere with the stability of the BB or the MtQ, these results demonstrate that KMP11 does not localize to the BB itself but to the TAC.

### KMP11 localizes to the α-helical region of p197

In line with the recovery of p197 in a KMP11 pulldown (Fig. 1C), the localization of KMP11 within the TAC best matches the cytosolic exclusion zone filaments consisting of p197 molecules (Gheiratmand et al., 2013; Aeschlimann et al., 2022). The short N- and the C-terminal domains of p197 connect to the mitochondrial outer membrane and the BB, respectively. The large central domain of p197 on the other hand determines the distance between the two structures. It consists of 35 or more α-helical repeats (175 aa each) predicted to form coiled-coil domains (Aeschlimann et al., 2022; Rabuffo et al., 2024).

**Fig. 3.**
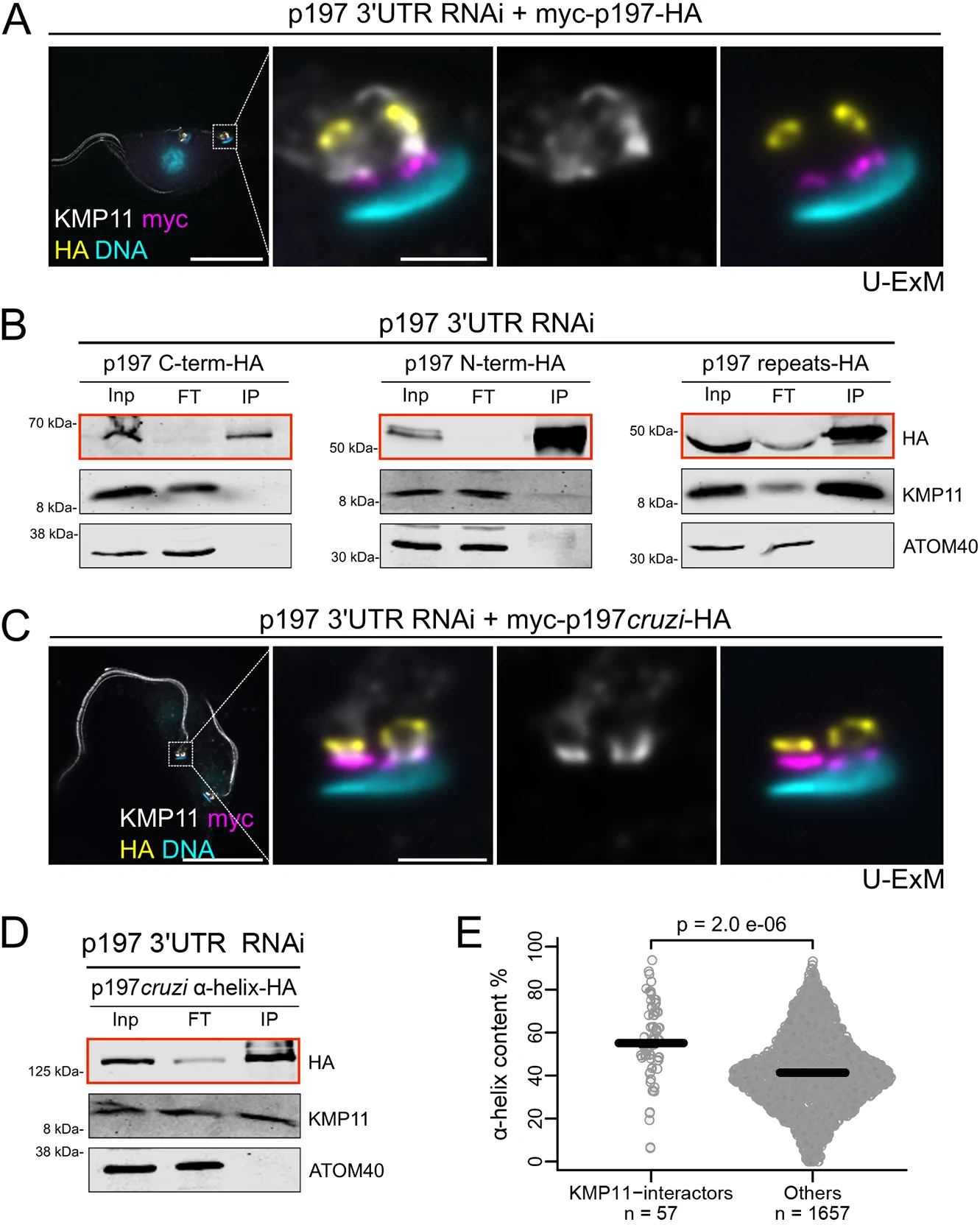
**KMP11 localizes to the α-helical region of p197.** (A) U-ExM of the p197 3′UTR RNAi cell line expressing N- and C-terminal tagged p197 (myc-p197-HA), both induced for 24 h. Cells stained for KMP11 (white), the tagged p197 with Myc (magenta) and HA (yellow), and for the DNA with DAPI (blue). Scale bars: 20 μm (left), 2 μm (enlargement). (B) Western blots from immunoprecipitations of different p197 constructs expressed in cells depleted of endogenous p197. Left, expression of the C-terminal domain of p197; middle, expression of the N-terminal domain of p197; right, expression of two repeat domains of p197. 10% of input (Inp) and flow through (FT) as well as 100% of the final eluate (IP) were analyzed by immunoblots probed with anti-HA antibody and anti KMP11 antibody. ATOM40 serves as negative control for the IP. (C) U-ExM of the p197 3′UTR RNAi cell line expressing N- and C-terminal tagged p197 where the α-helical domain of the *T. brucei* gene has been replaced by the α-helical domain from the *T. cruzi* p197 gene (myc-p197 *cruzi*-HA) induced for 24 h. Staining as in A. Scale bar: 20 μm (left), 2 μm (enlargement). (D) Same experiment as in B, but here the co-immunoprecipitation was performed with the Tet inducible p197 3′UTR RNAi cell line expressing the C-terminal HA-tagged α-helical domain of *T. cruzi* p197. Images in A–C are representative of hundreds of cells from at least three repeats. Blot in D representative of three repeats. (E) Quantification of the predicted α-helical content of KMP11-interacting versus non-interacting proteins. KMP11-interacting proteins were defined as all 57 proteins with a fold change of >5.0 (see Materials and Methods) in the KMP11–Myc co-immunoprecipitation dataset. The *P*-value of a two-tailed unpaired Student's *t*-test is indicated. In all U-ExM panels, the scale bars refer to the images as acquired (i.e. to the expanded specimen).

U-ExM and IF analysis of a cell line exclusively expressing a p197 variant containing distinct N- and C-terminal epitope tags demonstrated that KMP11 binds to the α-helical repeat domain of p197 (Aeschlimann et al., 2022) (Fig. 3A). This was confirmed by pulldown experiments using cell lines expressing truncated versions of *T. brucei* p197 consisting of either the N-terminal or C-terminal domains, or of two repeats of its central domain (Fig. 3B). In these experiments, only the central repeat domain of p197 was able to efficiently pull down KMP11 (Fig. 3B).

The same analysis was also undertaken for a cell line exclusively expressing an N- and C-terminally double tagged p197 variant in which the central repeat domain was replaced by the shorter α-helical central domain of its *Trypanosoma cruzi* ortholog (Fig. 3C). In a U-ExM and IF analysis, *T. brucei* KMP11 was localized to the p197 variant containing the central domain of the *T. cruzi* p197. As shown previously, exclusive expression of this variant resulted in a smaller BB–kDNA distance (Aeschlimann et al., 2022). Moreover, pulldown experiments using cell lines exclusively expressing the central domain of *T. cruzi* p197 confirmed that *T. brucei* KMP11 efficiently binds to this domain (Fig. 3D).

### Conclusions

*T. brucei* KMP11 has been reported to localize to the BB, flagellum and the FAZ (Li and Wang, 2008). Using IF and U-ExM, we confirmed its localization to the cytosolic FAZ filament. Moreover, we also found previously unknown localizations. The flagellum-associated KMP11 was present along the PFR and in the FC but was absent from the axoneme. We also found that KMP11 is not a BB protein but localizes to the central domain of p197 that forms the exclusion zone filaments of the TAC. Finally, we detected the protein at the MtQ and in the FPC/HC region. These localizations suggest that the majority of trypanosomal KMP11 does not need to directly interact with lipid bilayers, unlike the leishmanial KMP1. However, we cannot exclude that a small fraction of the KMP11 might associate also with lipids*.* Localization of ectopically expressed KMP11–Myc was consistent with the localization pattern of the endogenous protein (Fig. 1B; Fig. S2). The inability to detect KMP11–Myc at specific subcellular structures, including the MtQ and the FPC/HC region, is likely attributable to the comparatively low expression levels of the ectopically expressed protein in the presence of endogenous KMP11. Furthermore, analysis of tagged KMP11 localization in a KMP11 RNAi background was not feasible, as ectopic KMP11 expression levels were likely insufficient to compensate for depletion of the endogenous protein.

That a single protein binds to such diverse structures is unprecedented. A common feature of these localizations is that they represent a network of cytoskeletal structures, many of which have subunits with a high content of α-helices and coiled-coil domains (Wheeler et al., 2019; Gheiratmand et al., 2013). This suggests that KMP11 does not bind to a defined amino acid sequence but to a shared structural feature formed by α-helices. This is experimentally supported by the fact that *T. brucei* KMP11 interacts with the central domains of p197 from *T. brucei* and *T. cruzi*, respectively. Both domains, although not sharing any sequence similarity, have a high α-helical content and likely form coiled-coil domains (Aeschlimann et al., 2022). A further argument is that, among all detected proteins in the SILAC-IP pulled down by tagged KMP11, the proteins that are more than 5-fold enriched have on average a significantly higher α-helical content (55.1%) than the population that is less than 5-fold enriched (41.4%) (Fig. 3E). Thus, it is tempting to speculate that KMP11 might bind to α-helices in a rather promiscuous way and laterally crosslink them. The solved structure of KMP11 as an antiparallel homodimer would be consistent with such a function. In the case of the TAC, KMP11 might either form bundles between different p197 molecules, or, if the interaction would be intramolecular, it might function as stabilizer of coiled-coil domains. The proposed crosslinking function of KMP11 would be conceptually similar to the function of α-actinin, which crosslinks actin filaments (Sjoblom et al., 2008). Both proteins share the same quaternary structure – an antiparallel rod-shaped homodimer. However, α-actinin is, at 100 kDa, ∼9-fold larger than KMP11, suggesting that KMP11–p197 bundles would be much more closely spaced than the actin bundles formed by α-actinin. Moreover, at least in the case of p197, KMP11 would bundle mono-molecular filaments, whereas α-actinin crosslinks filaments that are formed by many globular actin molecules.

The kinetoplastid *Angomonas deanei* harbors a single bacterial endosymbiont, the division of which is coupled to its host cell. *A. deanei* KMP11 localizes to the BB and to fiber-like projections of the endosymbiont (Morales et al., 2023). However, the BB localization, as in *T. brucei*, might well represent a TAC localization – this raises the possibility that KMP11 links the TAC to the endosymbiont, possibly by recognizing an α-helical protein on its surface. Thus, the *A. deanei* TAC might mediate segregation of three single unit structures: the BB, the kDNA and the endosymbiont.

Presently, however, the functions of KMP11 we propose remain speculative. We do not know what features KMP11 recognizes in its multiple binding partners. Thus, our notion that KMP11 is a crosslinking protein should serve as a working hypothesis that can be tested by future studies.

## MATERIALS AND METHODS

### Cell lines

The procyclic *T. brucei* cell lines, except for the KMP11–Myc line, were derived from the single-marker inducible 427 strain (Aeschlimann et al., 2022). Cells were maintained at 27°C in SDM-79 medium supplemented with 5% (v/v) fetal calf serum (FCS). The KMP11–Myc cell line used for mass spectrometry was derived from the 29-13 strain and grown under the same conditions but with 10% FCS.

KMP11 (Tb927.9.13820) was C-terminally tagged with a triple HA or Myc tag using the inducible pLew100 vector system (Wirtz et al., 1999). The open reading frame (ORF) was amplified by PCR using primers (F) 5′-ACATTAAAGCTTATGGCCACCACATACGAAGA-3′ and (R) 5′-CGTATTGGATCCTTTTCCGGGGAACTGGGCAT-3′. The PCR product was cloned into a modified pLew100 vector, which contains a puromycin resistance gene as well as a C-terminal triple epitope tag.

For KMP11 expressed in an RNAi background, a synthetic gene was ordered from Biomatik with an altered DNA sequence, rendering the corresponding mRNA resistant to the RNAi while encoding for the same amino acid sequence. The sequence of RNAi resistant KMP11 is: 5′-ATGGCAACCACGTACGAGGAATTCGCTGCAAAGCTTGACCGTCTCGACGCCGAGTTCGCAAAGAAAATGGAAGAGCAAAACAAACGATTTTTCGCAGACAAACCTGACGAGGCAACGTTGTCCCCAGAAATGAAGGAGCATTATGAGAAGTTTGAAAAGATGATTCAGGAACACACAGACAAATTCAATAAGAAAATGCGTGAGCATTCAGAACACTTTAAGGCAAAGTTCGCGGAGCTCCTTGAGCAACAGAAAAATGCACAGTTTCCCGGCAAATGA-3′.

This recoded ORF was amplified by PCR using primers (F) 5′-ACATTACATATGATGGCAACCACGTACGAGGA-3′ and (R) 5′-AGTATTCTCGAGTTTGCCGGGAAACTGTGCAT-3′. The PCR product was cloned into a modified pLew100 vector, which contains a puromycin resistance gene as well as a C-terminal triple HA tag.

The inducible pLew100 vector system was also used to generate C-terminally HA-tagged constructs of the p197 N-terminus, C-terminus, and α-helical regions from both *T. brucei* (2 repeats) and *T. cruzi* (whole α-helical region). The p197 3′UTR RNAi cell line, the double-tagged p197 cell line (myc-p197-HA), and the inducible double-tagged version containing the *T. cruzi* α-helical domain (myc-p197*cruzi*-HA) have been described previously (Aeschlimann et al., 2022).

The KMP11-RNAi cell line was prepared using a pLew100-derived vector containing a blasticidin resistance gene, with the generation of a stem–loop construct occurring by the insertion of the RNAi inserts in opposing directions. The loop is formed by a 460 bp spacer fragment. RNAi plasmids were prepared targeting KMP11 via its ORF. PCR was used to amplify the RNAi targets with primers (F) 5′-ACATTAAAGCTTGGATCCATGGCCACCACATACGAAGA-3′ and (R) 5′-CGTATTTCTAGACTCGAGTCATTTTCCGGGGAACTGGG-3′. RNAi efficiency was verified by RNA extraction and northern blotting. RNA of uninduced and 2 days induced cells was isolated via acid guanidinium thiocyanate-phenol-chloroform extraction. The RNA samples were separated on a 1% agarose gel in 20 mM MOPS buffer supplemented with 0.5% formaldehyde. Radioactive probes were generated from the gel-purified RNAi insert (mentioned above) and radioactively labelled with the Prime-a-Gene labelling kit (Promega).

### SILAC labelling and co-immunoprecipitation for mass spectrometry

Cells expressing KMP11–Myc were grown in SDM80 medium supplemented with either light (^12^C_6_/^14^N) or heavy (^13^C_6_/^18^N) isotopes of arginine (1.1 mM) and lysine (0.4 mM) (Euroisotop), along with 10% dialyzed FCS (BioConcept, Switzerland). To ensure full incorporation of labeled amino acids, cells were cultured in SILAC medium for 5 days. At 16 h before harvesting, KMP11–Myc expression was induced in either the light or heavy condition (one light-induced, two heavy-induced) by treatment with 1 μg/ml tetracycline (Thermo Fisher Scientific).

A total of 4×10^8^ cells were mixed, harvested (2500 ***g*** for 8 min) and lysed for 15 min on ice in lysis buffer [20 mM Tris-HCl pH 7.4, 0.1 mM EDTA, 100 mM NaCl, 10% glycerol, 1% digitonin, and EDTA-free protease inhibitors (Roche, #04693116001)]. Lysates were cleared by centrifugation (16,000 ***g***, 4°C, 15 min), and supernatants were incubated for 1 h at 4°C with 80 μl anti-Myc affinity beads (Roche), pre-washed in wash buffer (lysis buffer containing 0.1% digitonin). After incubation, beads were washed three times with 500 μl wash buffer, and bound proteins were eluted by boiling for 5 min in 0.1% SDS in 60 mM Tris-HCl pH 6.8.

### Quantitative mass spectrometry

Eluates obtained in KMP11 SILAC-IP experiments (*n*=3 independent replicates) were loaded onto 4–12% NuPAGE Bis-Tris gradient gels (Life Technologies). Electrophoresis was stopped after the proteins had migrated ∼1 cm into the gel and proteins were stained with colloidal Coomassie Blue. The protein-containing regions of the gel were excised and cut into smaller pieces (Dewar et al., 2022). Cysteine residues were subsequently reduced with 5 mM TCEP (Thermo Fisher Scientific, #77720) and alkylated with 100 mM iodoacetamide (Sigma-Aldrich, #16125), followed by tryptic in-gel digestion as described previously (Peikert et al., 2017). Tryptic peptides were analyzed by liquid chromatography-mass spectrometry (LC-MS) on an Orbitrap Elite MS instrument (Thermo Fisher Scientific) as described before (Dewar et al., 2022).

Protein identification and SILAC-based relative quantification were carried out using MaxQuant/Andromeda (version 1.6.5.0; Tyanova et al., 2016). Database searches were conducted against the *T. brucei* TREU927 proteome downloaded from the TriTryp database (version 8.1). Data analysis was performed with tryptic specificity, allowing up to two missed cleavages. Mass tolerances for precursor and fragment ions were set to 4.5 ppm and 0.5 Da, respectively. Carbamidomethylation of cysteine residues was specified as fixed modification, whereas N-terminal acetylation and oxidation of methionine were included as variable modifications. Arg10 and Lys8 were defined as heavy labels. Protein identification was based on the assignment of at least one unique peptide of at least seven amino acids. The false discovery rate applied to both lists of peptide and protein identifications was set to 0.01. The feature ‘requantify’ was enabled. Relative protein abundance ratios were determined based on unique peptides and at least one SILAC peptide pair. The mean log_10_ of normalize with and without tetracycline (Tet) (KMP11–Myc and control) ratios was calculated, and *P*-values were determined using a one-sided Student's *t*-test. For information about identified and quantified proteins, see Table S2.

### Co-immunoprecipitation and western blot analysis

A total of 2×10^8^ cells from an overnight-p197 3′UTR RNAi-induced culture inducibly expressing the different HA constructs were harvested, washed in phosphate-buffered saline (PBS), and lysed in RIPA buffer [50 mM Tris-HCl pH 8.0, 150 mM NaCl, 0.5% Triton X-100, 0.5% deoxycholate, 0.1% SDS, supplemented with 1× protease inhibitor mix (cOmplete protease inhibitor complex; Roche)]. Cells were passed 10 times through a needle and incubated on ice for 15 min. Lysates were cleared by centrifugation (16,000 ***g***, 15 min, 4°C), and the supernatant was incubated with 50 μl anti-HA affinity beads (Roche) pre-washed in RIPA buffer (see above).

After 1 h of incubation at 4°C on a rotating wheel, a sample of the unbound fraction (FT) was taken. Beads were washed six times with 1 ml RIPA buffer and then boiled in sample loading buffer (without β-mercaptoethanol). For SDS-PAGE and western blot analysis, 10% of the input (INP), 10% of the flow-through (FT) and 100% of the eluate (IP) were loaded. Uncropped images of blots from this paper can be seen in Fig. S4.

### Immunofluorescence microscopy

10^6^ cells were harvested (2500 ***g*** for 8 min), washed resuspended in PBS, and allowed to settle on coverslips for 10 min. Then, cells were fixed in 4% paraformaldehyde (PFA) for 10 min and washed in PBS. Subsequently, cells were treated with 0.05% Triton X-100 for 5 min and washed again in PBS. Cells were then blocked in 4% bovine serum albumin (BSA) and primary and secondary antibodies were diluted in PBS containing 2% BSA. After staining, cells were washed in PBS and mounted onto a 10 μl drop of Vectashield with DAPI (Vectorlabs).

Images were acquired using a DMI6000B microscope equipped with a DFC360 FX monochrome camera and LAS X software (Leica Microsystems). Further image processing and analysis were done using Fiji software with the FigureJ plugin.

### Ultrastructure expansion microscopy

2×10^6^ cells were centrifuged for 3 min at 2000 ***g***, washed twice with 1 ml PBS, and resuspended in 500 μl PBS. From this, 50 μl were settled for 10 min on 12 mm poly-D-lysine-coated coverslips (Menzel-Gläser). Coverslips were transferred to a 24-well plate containing FA/AA solution [0.7% formaldehyde (36.5–38%, Sigma) and 1% acrylamide (40%, Sigma) in PBS] and incubated for 5 h at 37°C.

A humidified plastic chamber was prepared with wet tissue and parafilm and placed on ice. A 35 μl drop of monomer solution [19% sodium acrylate (AK Scientific, 7446-81-3), 10% acrylamide, 0.1% N,N′-methylenebisacrylamide (Sigma) in PBS, with 0.5% ammonium persulfate and 0.5% TEMED] was placed on the parafilm. Coverslips were carefully placed cell-side down onto the drop. Gelation proceeded for 5 min on ice, followed by 1 h at 37°C.

Gels were transferred to a 6-well plate with 2 ml denaturation buffer (200 mM SDS, 200 mM NaCl, 50 mM Tris-HCl pH 9.0 in water) for 20 min at room temperature to detach from coverslips. Detached gels were then moved to 1.5 ml tubes with fresh denaturation buffer and incubated at 95°C for 1 h.

After denaturation, gels were placed in deionized water for initial expansion. Water was replaced after 30 min, and gels were incubated overnight in fresh deionized water. The next day, gels were washed twice in PBS (15 min each), before adding antibodies.

### Antibodies

Polyclonal rabbit antiserum against TbKMP11 was commercially produced (Eurogentec, Belgium, 87 days; immunization of the two animals with the mix of coupled peptides) using aa 26–40 (QNKRFFADKPDEATL), and aa 78–92 (AELLEQQKNAQFPGK) as antigens. The peptides were suggested by the company and, according to AlphaFold prediction, they localize in the helix kink and at the very C-terminus. For both peptides, 5 ml of serum was purified against the peptide.

In this study, the following antibodies were used: polyclonal rabbit antiserum against TbKMP11 [1:500 for IF and U-ExM, 1:100 in western blotting (WB)]; rat monoclonal α-tubulin YL1/2 (1:2000 in IF); monoclonal mouse anti-acetylated α-tubulin (6-11B-1, Santa Cruz Biotechnology, 1:500 in U-ExM); anti-c-Myc monoclonal Antibody, mouse (Thermo Fisher Scientific, 13-2500, 1:500 in U-ExM); rat anti-HA, High Affinity (Sigma, 11867423001, 1:500 in U-ExM, 1:5000 in WB); polyclonal rabbit antiserum against ATOM40 (1:10000 In WB); goat anti-mouse IgG (H+L) highly cross-adsorbed secondary antibody, Alexa Fluor™ 488 (Thermo Fisher Scientific, A32723, 1:500 in U-ExM); anti-rabbit-IgG conjugated to ATTO 647 (Sigma, #40839, 1:500 in U-ExM); donkey anti-rat IgG (H+L) highly cross-adsorbed secondary antibody, Alexa Fluor™ 594 (Thermo Fisher Scientific, A21209, 1:500 in U- ExM), goat anti-rabbit IgG (H+L) antibody, Alexa Fluor™ 594 (Thermo Fisher Scientific, A32723, 1:1000 in IF), goat anti-rat IgG (H+L) antibody, Alexa Fluor™ 488 (Thermo Fisher Scientific, A32723, 1:1000 in IF).

### Imaging and image analysis

After expansion, the gel was cut and mounted onto poly-D-lysine-coated 25 mm glass-bottom dishes (D35-20-1.5-N, Cellvis; 35 mm dish with 20 mm microwell and #1.5 cover glass). *Z*-stacks were acquired on a Nikon Ti2 Kinetix Widefield Microscope equipped with a Photometrics Kinetix 500 fps camera, using a 100× objective (NA=1.45). Imaging was performed with a *z*-step size of 0.2 μm and a pixel size of 65 nm. Images were deconvolved using Huygens HRM software and further analyzed in ImageJ.

### Annotation of the proteomic dataset

Proteins that were more than 1.5-fold significantly enriched (*P*<0.05) in the proteomic dataset from the KMP11–Myc immunoprecipitation (IP) were further annotated according to the protein's localization (Sunter et al., 2023) and/or their respective function listed on TriTrypDB (https://tritrypdb.org/tritrypdb/app), and accordingly this color code was used in Fig. 1C and Table S1.

We defined candidate KMP-interacting proteins using an enrichment cutoff of >5-fold in the SILAC-based co-immunoprecipitation data. This threshold was fixed before and independently of any downstream analysis, including the assessment of predicted α-helical content. Two considerations motivated the choice. First, a >5-fold ratio is a deliberately stringent, conservative criterion for quantitative SILAC-CoIP experiments: whereas 2-fold enrichment is often treated as significant in such datasets, a 5-fold cutoff substantially lowers the expected false-positive rate and selects for high-confidence interactors, at the acknowledged cost of sensitivity. Second, and importantly, because the cutoff was applied solely to the co-IP enrichment values - with no reference to the structural features tested later, the subsequently observed increase in α-helical propensity cannot be an artifact of how the interacting set was defined.

### Calculation of the α-helical contents of KMP11 interactors

The PASTA 2.0 Algorithm was used to calculate the α-helical contents of the individual proteins identified within the KMP11–Myc SILAC IP MS data set (Walsh et al., 2014).

## Supplementary Material



10.1242/joces.264898_sup1 Supplementary information

Table S2. Identification of KMP11-interacting proteins using SILAC proteomics
