# Supplementary material for: From the flagellum attachment zone to the tripartite attachment complex – widespread cytoskeletal engagement of T. brucei KMP11
Source: J Cell Sci. 2026 Aug 20;139(21):jcs264898. doi: 10.1242/jcs.264898 (PMC13539426; doi:10.1242/jcs.264898)
Supplement: Supplementary file 1 [file joces-139-264898-s1.pdf]

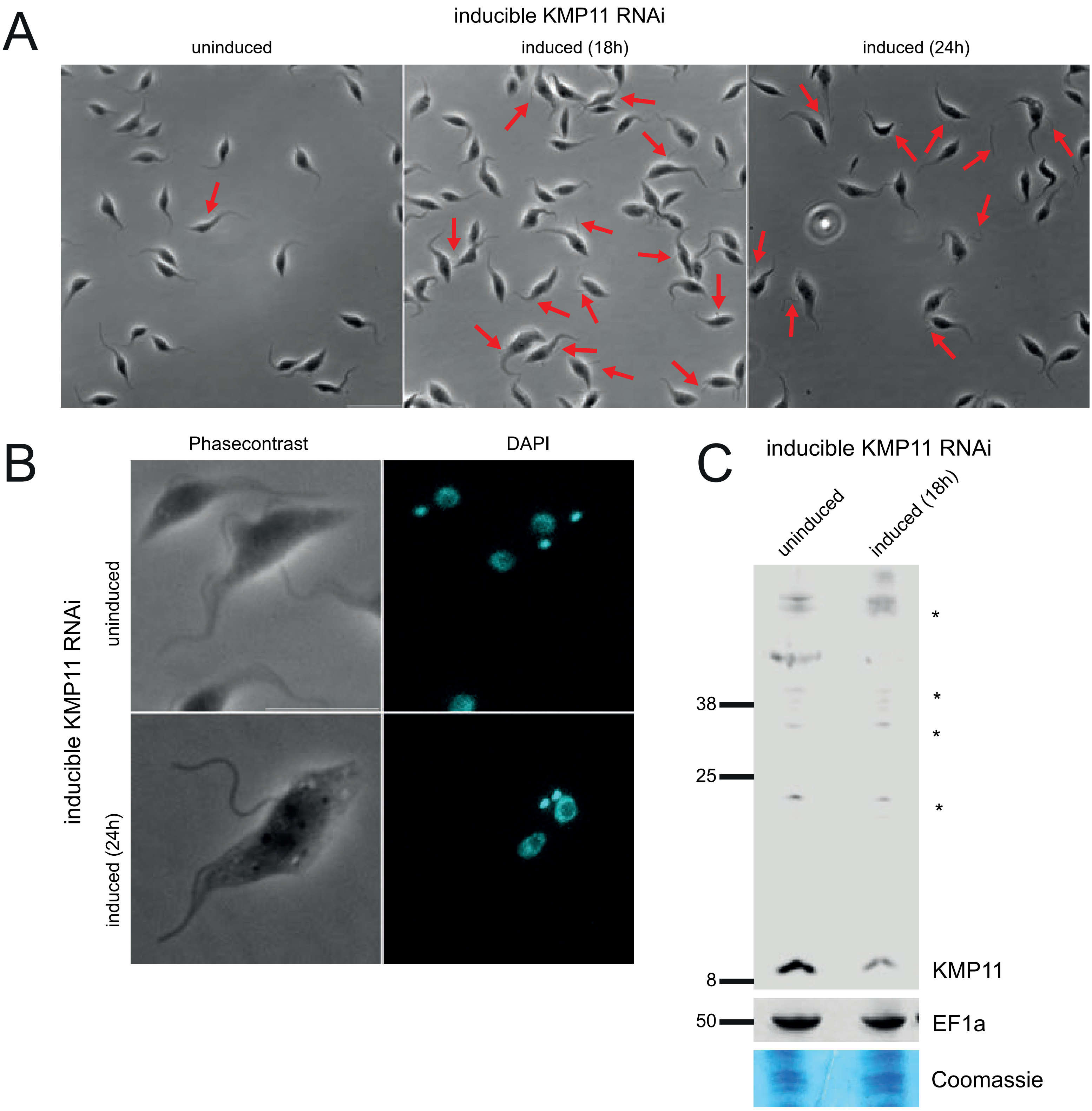

Fig. S1. Induction of KMP11 RNAi in procyclic-form *T. brucei* results in detachment of the new flagellum and depletion of a 11 kDa protein on western blot. A) Phase-contrast microscopy of procyclic-form cells carrying an inducible KMP11 RNAi construct cultured without tetracycline (uninduced) or following RNAi induction for 18 h and 24 h. Induced cells display pronounced detachment of the new flagellum (red arrows). Scale bar = 20µm. **B)** Representative phase-contrast and DAPI fluorescence images of uninduced and KMP11 RNAi-induced 2K2N cells (24 h). Scale bar = 10µm. **C)** Western blot analysis of KMP11 protein levels in uninduced cells and after 18h of RNAi induction. EF1a served as a loading control and Coomassie staining is shown as an additional loading control. Molecular weight markers (kDa) are indicated on the left. * indicate nonspecific signals.

Journal of Cell Science • Supplementary information

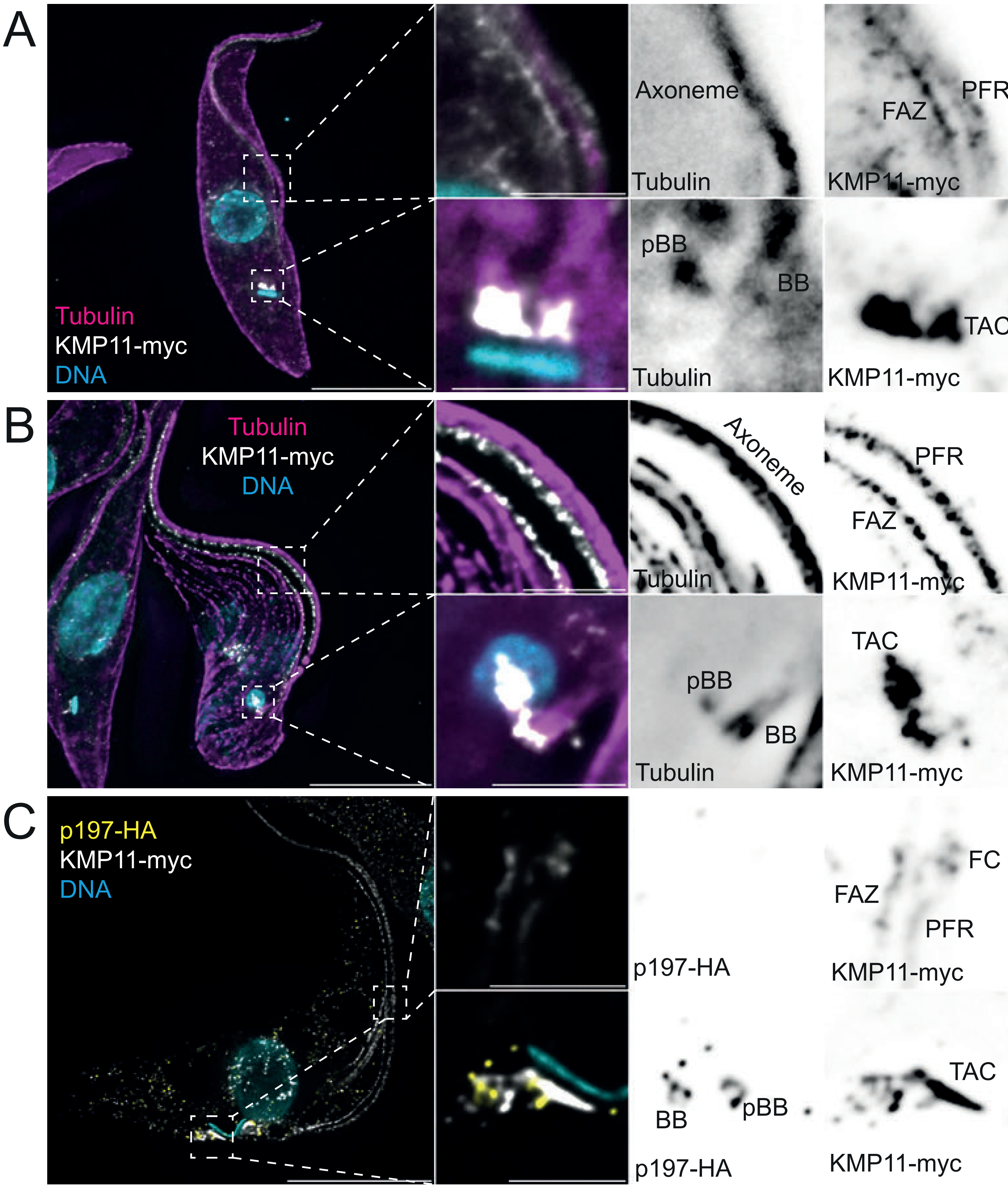

Fig. S2. U-ExM of KMP11-myc stained with anti-myc antibodies. A, B) U-ExM of PCF cells ectopically expressing KMP11-myc stained for KMP11-myc (white), 6-11B-1 antibody for α-tubulin (magenta) and DAPI for the DNA (cyan). Scale bar 10 μm (left), 2 μm (enlargement). C) U-ExM of PCF cells ectopically expressing KMP11-myc and p197-HA stained for KMP11-myc (white), p197-HA (yellow) and DAPI for the DNA (cyan). Scale bar 10 μm (left), 2 μm (enlargement). Abbreviations: FC, flagella connector; PFR, paraflagellar rod; FAZ, flagellum attachment zone; TAC, tripartite attachment complex.

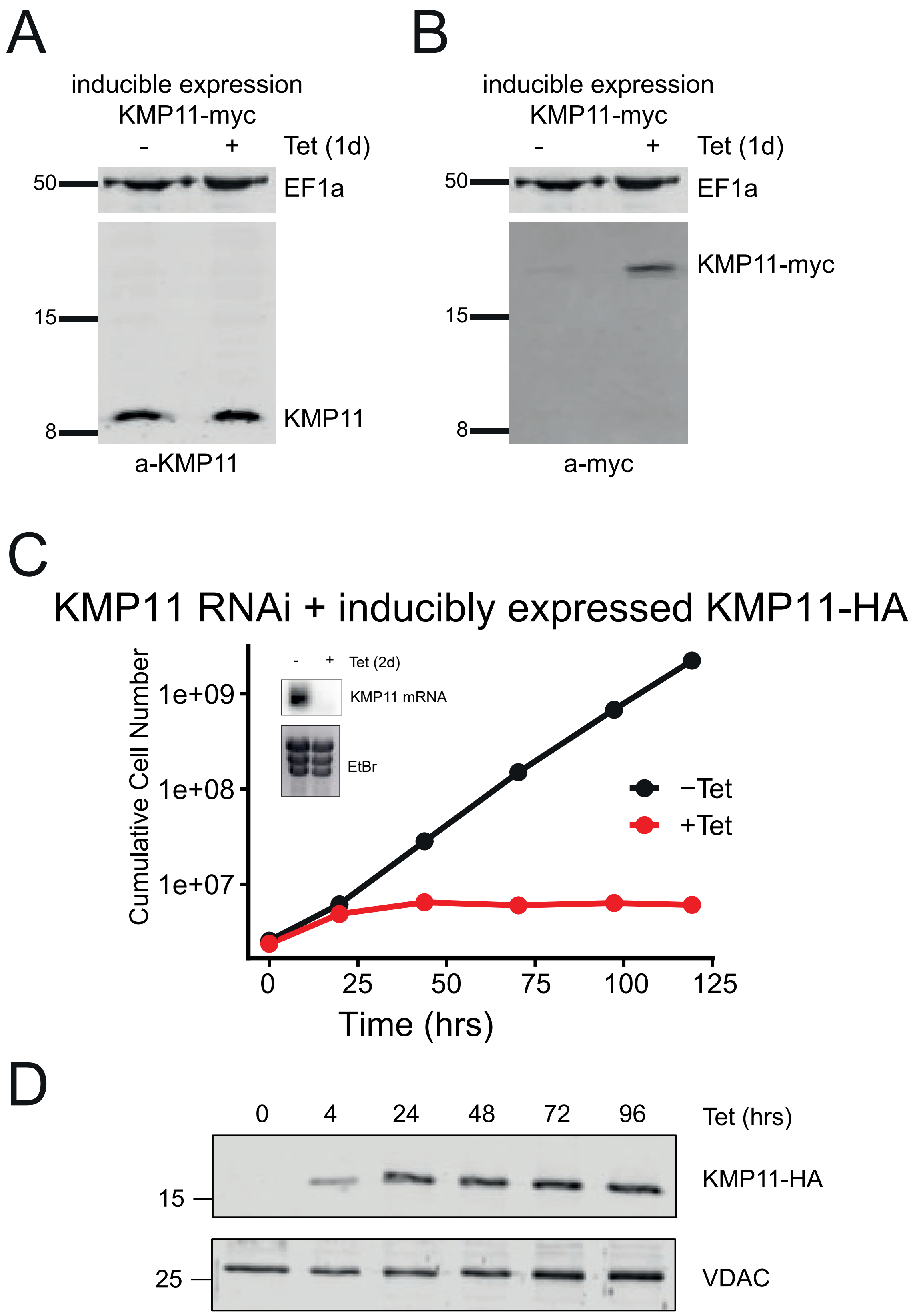

Fig. S3. Endogenous KMP11 is more abundant than ectopically expressed KMP11. A, B) Western blot analysis of uninduced and one day induced KMP11-myc expressing PCF trypanosomes. The membrane was decorated (A) first with serum against KMP11 (B) subsequently with anti-myc. EF1alpha serves as loading control. Molecular-weight markers (kDa) are indicated on the left. **C)** Growth curve of PCF cells expressing KMP11-HA as well as KMP11-RNAi. Inset shows a Northern blot analysis of isolated RNA extracted from uninduced and two days induced cells, probed for KMP11 mRNA. Ethidium bromide serves as a loading control. **D)** Western blot analysis of whole cell lysates from indicated timepoints post induction. Molecular weight markers (kDa) are indicated on the left. The membranes were probed for HA; VDAC serves as a loading control.

Journal of Cell Science • Supplementary information

Table S1. KMP11 interacting proteins.

acc. number	annot. name	TrypTag	fold enrichment
Tb927.1.4310	FAZ2	Flagella	12.6
Tb927.9.13820	KMP11	Flagella / BB	11.6
Tb927.10.15750	p197	TAC	11.2
Tb927.1.5000			11.1
Tb927.10.14220		Flagella	11.0
Tb927.11.8370	NuSAP1	Nucleus	10.6
Tb927.2.5660	ADKA	Flagella	10.1
Tb927.3.5310	PFR	Flagella	10.0
Tb927.4.2080	CC2D	FAZ	10.0
Tb927.9.4450		Cytoplasm	9.8
Tb927.4.2300		Flagella	9.7
Tb927.10.8070	ATPase+C16	Mitochondrion / kDNA	9.2
Tb927.3.2490		Flagellar pocket	9.1
Tb927.4.4090		Golgi	8.9
Tb927.11.6430		Cytoplasm	8.9
Tb927.11.14970		Flagella	8.8
Tb927.11.3110		Flagella	8.8
Tb927.6.1730			8.7
Tb927.4.2220	DnaJ	BB region	8.7
Tb927.11.14230			8.5
Tb927.10.830	Kinase	Flagella	7.9
Tb927.10.4060		Cytoplasm / Nucleus	7.9
Tb927.6.1430		Cytoplasm	7.7
Tb927.11.2030			7.6
Tb927.10.15400	Kinesin	Cytoplasm	7.5
Tb927.11.16670		Cytoplasm	7.4
Tb927.9.15470	Kinesin	Cytoplasm / Hook complex	7.4
Tb927.10.9950		Cytoplasm	7.4
Tb927.11.11280		Flagella	7.3
Tb927.4.4670		Cytoplasm	6.9
Tb927.11.6540		Golgi	6.6
Tb927.10.6480		Cytoplasm	6.4
Tb927.9.15260		Cytoplasm / Nucleus	6.3
Tb927.9.9730	FP45	Flagellar pocket	6.3
Tb927.7.3710		Cytoplasm / Nucleus	6.2
Tb927.11.8180			6.1
Tb927.11.12530	FAZ3	FAZ	6.0
Tb927.8.2860		Cytoplasm	5.9
Tb927.8.1210		Cytoplasm	5.9
Tb927.4.3740	FAZ1	FAZ	5.8
Tb927.11.13980	ODA7	Cytoplasm	5.5
Tb927.9.1780	SLY1	Golgi	5.5
Tb927.8.6980	FAZ14	FAZ	5.4
Tb927.11.10740			5.4
Tb927.4.1730	NuSAP4	Nucleus	5.2
Tb927.11.13670		Flagella / Cytoplasm	5.2
Tb927.6.4550		Cytoplasm / Nucleus	5.2
Tb927.4.640		Golgi	5.1
Tb927.11.15100	Tb5.20	Flagella	5.0
Tb927.10.4490		Flagella	5.0
Tb927.9.5700		Golgi	4.9
Tb927.11.2020		Cytoplasm / Flagella	4.9
Tb927.5.4520	KKIP1	Kinetochore	4.8
Tb927.7.5500		Cytoplasm	4.5
Tb927.8.4940		Cytoplasm / Flagellar pocket	4.3
Tb927.4.850		Flagella	4.3
Tb927.11.3050		BB	4.0
Tb927.9.10530	FAZ4	FAZ	3.9
Tb927.6.580		Golgi	3.9
Tb927.11.3500		Flagella	3.8
Tb927.11.11210	PFC17	PFR	3.7
Tb927.4.2060	FAZ8	FAZ	3.6
Tb927.4.310		Cytoplasm	3.3
Tb927.8.4950	Kinesin	Flagellar pocket	3.2
Tb927.9.7690		Cytoplasm	3.1
Tb927.9.5040	PDEB1	Cytoplasm / Flagella	2.7
Tb927.11.6270		Nucleus	2.5
Tb927.3.930	Dynein		2.4
Tb927.8.6320		Cytoplasm	2.3
Tb927.7.3410	Centrin4	BB	2.3
Tb927.8.1870	tGLP1	Golgi	2.2
Tb927.3.3030		Flagellar pocket	1.9
Tb927.10.14320	FAZ9	FAZ	1.8
Tb927.10.1450	CAAP1	BB	1.7
Tb927.7.2330		Nuclear envelope	1.7
Tb927.8.2300		Mitochondrion	1.6
Tb927.1.4420		FAZ / ER	1.6
Tb927.10.16150			1.6
Tb927.11.5290	MCP9	Mitochondrion	1.5
Tb927.11.9400		Flagella	1.5
Tb927.6.3570		Mitochondrion / kDNA	1.5
Tb927.4.2070		Cytoplasm / Cytoskeleton	1.5

Proteins that were found more than 1.5-fold significantly enriched (p-value < 0.05) in the proteomic dataset from the KMP11-myc SILAC IP were further annotated according to the protein's localization [34] and/or their respective function listed on TriTrypDB (https://tritrypdb.org/tritrypdb/app). Blue: flagella, flagellar attachment zone (FAZ) and paraflagellar rod (PFR), Green: flagellar pocket region including basal body (BB), microtubule quartet (MtQ) and flagellar pocket collar / hook complex (FPC/HC), Yellow: nucleus. The colour code in the volcano plot in Fig 1C was matched accordingly.

Table S2. Identification of KMP11-interacting proteins using SILAC proteomics

Available for download at
https://journals.biologists.com/jcs/article-lookup/doi/10.1242/jcs.264898#supplementary-data

Journal of Cell Science • Supplementary information

B
Fig. 3

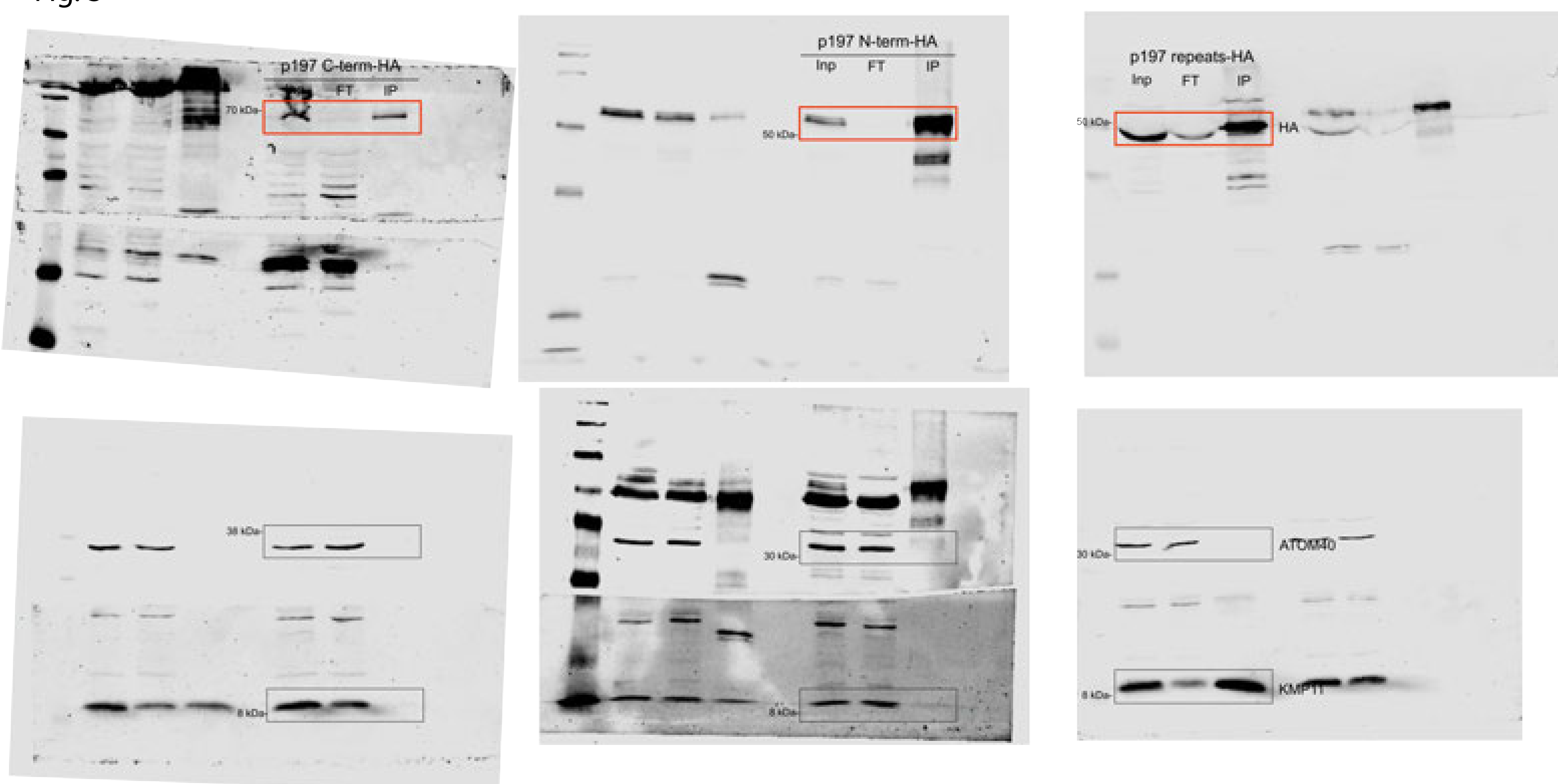

D

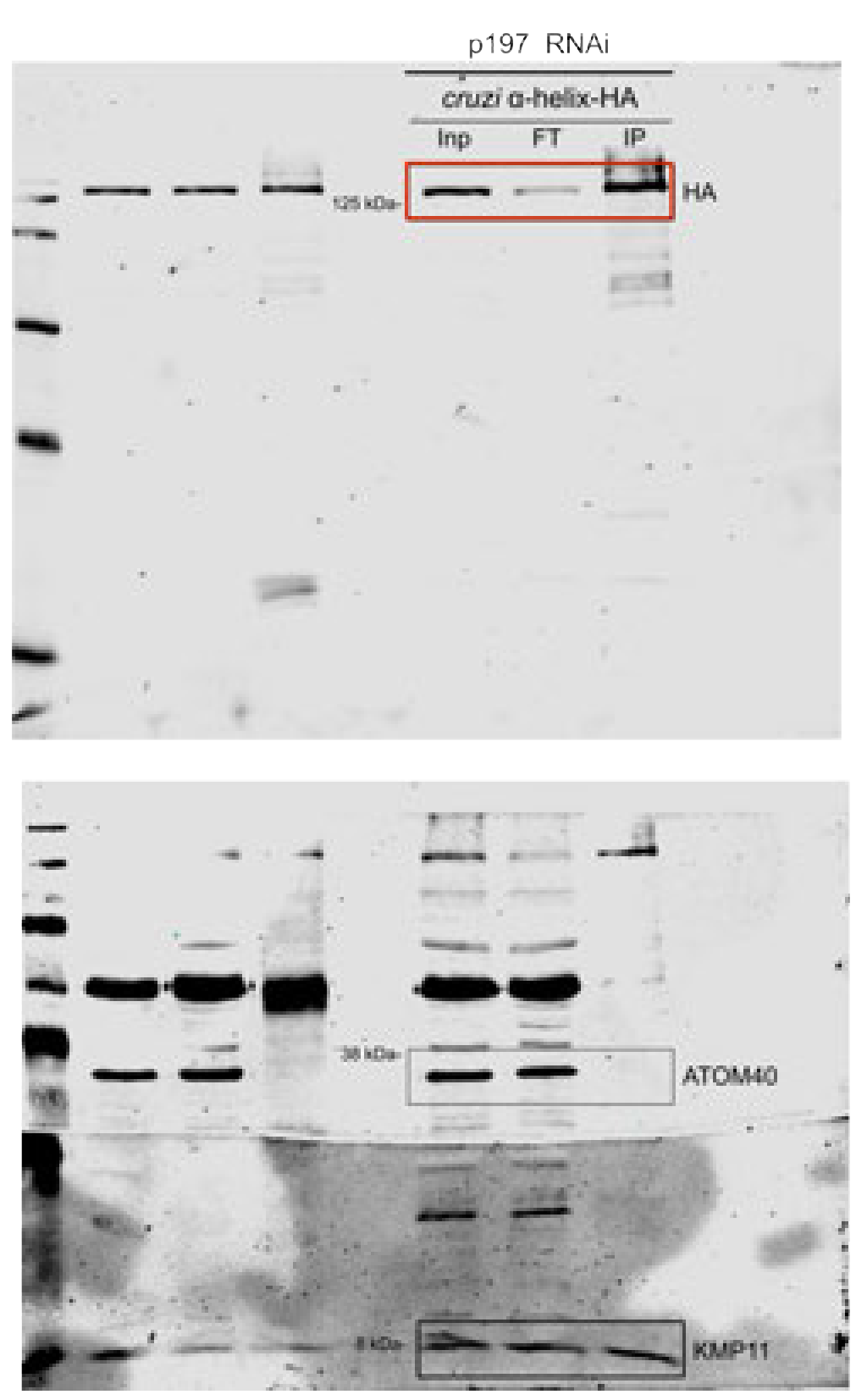

Fig. S1
C

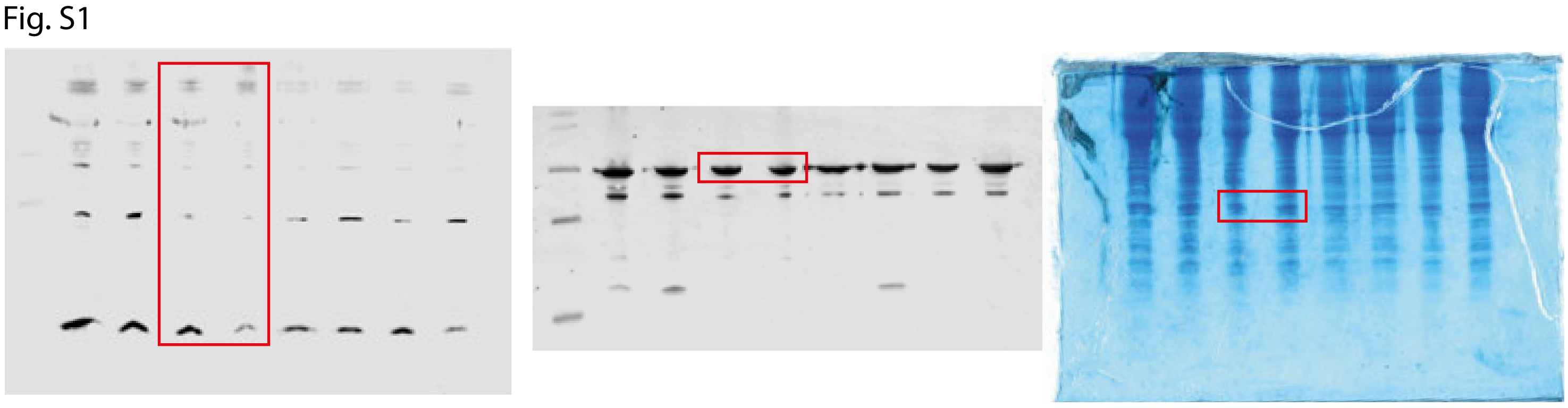

Journal of Cell Science • Supplementary information

Fig. S3

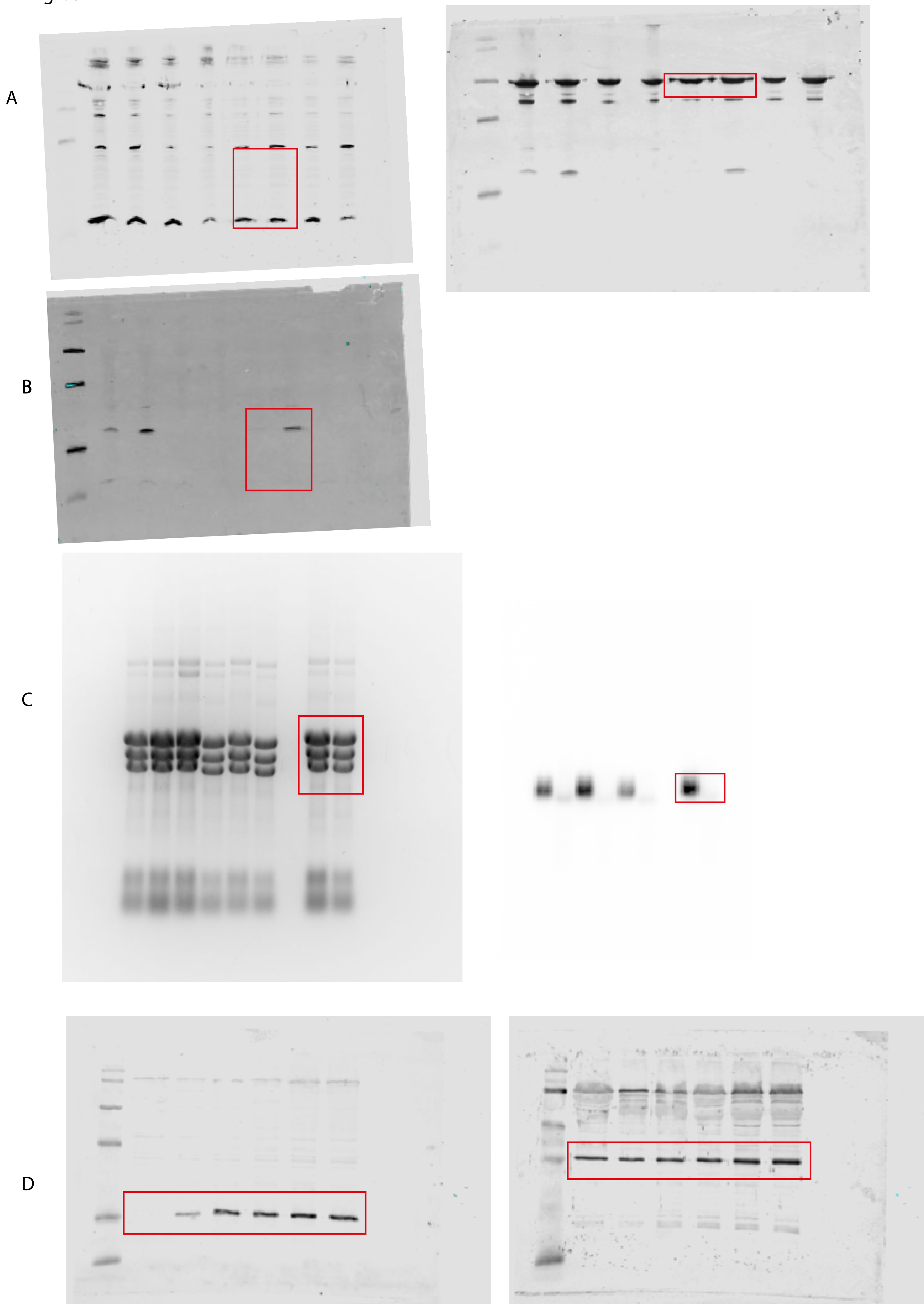

Fig. S4. Blot transparency

Journal of Cell Science • Supplementary information
