# Supplementary material for: From the flagellum attachment zone to the tripartite attachment complex – widespread cytoskeletal engagement of T. brucei KMP11
Source: J Cell Sci. 2026 Aug 20;139(21):jcs264898. doi: 10.1242/jcs.264898 (PMC13539426; doi:10.1242/jcs.264898)
Supplement: Supplementary file 2 [file joces-139-264898_review_history.pdf]

Journal of Cell Science

# From the FAZ to the TAC: Widespread Cytoskeletal Engagement of T. brucei KMP11

Salome Aeschlimann, Clirim Jetishi, Caroline E. Dewar, Philip Stettler, Markus Gerber, Silke Oljeklaus, Bettina Warscheid, André Schneider and Torsten Ochsenreiter
DOI: 10.1242/jcs.264898

**Editor**: Michael Way

**Review timeline**
Original submission: 20 March 2026
Editorial decision: 15 April 2026
First revision received: 22 May 2026
Accepted: 13 June 2026

---

**Original submission**

First decision letter

MS ID#: jcs.264898

MS TITLE: From the FAZ to the TAC: Widespread Cytoskeletal Engagement of T. brucei KMP11

AUTHORS: Salome Aeschlimann; Clirim Jetishi; Caroline E. Dewar; Philip Stettler; Markus Gerber; Silke Oljeklaus; Bettina Warscheid; Torsten Ochsenreiter; AndrÃ© Schneider

ARTICLE TYPE: Short Report

Dear Dr Ochsenreiter,

We have now reached a decision on the above manuscript.

As you will see, the reviewers raise a number of criticisms that prevent me from accepting the paper at this stage. They suggest, however, that a revised version might prove acceptable, if you can address their concerns including demonstrating the specificity of the KMP11 antibody. If you think that you can deal satisfactorily with the criticisms on revision, I would be pleased to see a revised manuscript.

Reviewer 1

SUMMARY OF THE ADVANCE MADE IN THIS PAPER AND ITS POTENTIAL SIGNIFICANCE TO THE FIELD

In this short report, Aeschlimann et al. generated a polyclonal antibody against T. brucei KMP11, a kinetoplastid specific protein with diverse cellular distribution and essential functions, yet unclear mechanisms. Using this antibody, the authors reported KMP11 association with TAC, MtQ, HC and FC but not basal bodies. Based on previously characterized KPM11 interactions and SILAC-IP performed in this study, the authors proposed that KMP11 might bind promiscuously to cytoskeletal proteins containing alpha helices, thus explaining their widespread distribution to multiple cytoskeletal elements. A crosslinking function of KMP11 is also speculated.

SUGGESTIONS TO AUTHORS

© 2026. Published by The Company of Biologists under the terms of the Creative Commons Attribution License (https://creativecommons.org/licenses/by/4.0/).

Major comments

1. I could not find any validation of the specificity of the anti-KMP11 antibody used in this study. As several new cellular localizations of KMP11 are reported using this antibody, control for antibody specificity is essential. This antibody was used for IF, U-ExM as well as Western blots, validation of its specificity to KMP11 (e.g. loss of signal upon KMP11-RNAi, purified KMP11 protein for competition analyses etc) need to be shown for all above experimental conditions.

2. The proposed model that KMP11 interacts promiscuously with alpha helixes is interesting, but lacks strong experimental support. The interaction between KMP11 and p197 central helical region is shown by co-IP. The model would be stronger if supported by direct binding using purified components. The cross-species result (binding to T. cruzi p197 central domain) is interesting, but it still doesn't exclude the presence of "bridging proteins" conserved across trypanosomes.

3. Even for a short report, I think the speculated crosslinking function of KMP11 requires some test. Specifically, whether KMP11 depletion affects the organization/spacing/filament properties of p197 or other cytoskeletal proteins, in vivo or in vitro?

4. KMP11 was first reported in Leishmania as a membrane protein. To my knowledge, its membrane association is not tested in T. brucei, even though purified KMP11 forms structured 4-helix bundle in DPC micelles. While the proposed model and discussion focused on KMP11 binding to various cytoskeletal elements, its membrane association, even possible membrane-cytoskeleton crosslinking shall not be overlooked.

Minor comments

The SILAC-IP study was performed on cells with tetracycline-induced KMP11-myc expression (from pLEW100). Characterization of this cell line is not shown. Importantly, how much is KMP11-myc overexpression compared to native KMP11? Does overexpressed KMP11-myc show the same cellular localizations as native KMP11? These are important, considering the potentially promiscuous nature of KMP11 binding.

For both KMP11-myc and native KMP11, how soluble are these proteins? The cells were solubilized with 1% digitonin in the SILAC-IP experiments. How much KMP11 remains insoluble under this condition? Focusing on the soluble fraction for coIP in this study is OK, but I am afraid most (and some of the strongest) KMP interactions may be overlooked in the insoluble fractions.

<u>Reviewer 2</u>

MS ID#: jcs.264898 MS Title: From the FAZ to the TAC: Widespread Cytoskeletal Engagement

Authors: Aeschlimann et al

This interesting manuscript revisits the localisation of KMP11, a unique Kinetoplastid protein which has puzzled the community since its discovery because of its localisation to multiple structures. The authors generated a new antibody (targeting a couple of KMP11 peptides) and using Ultrastructure Expansion Microscopy (U-ExM), they refine KMP11 location at the flagellum level (paraflagellar rod - PFR - rather than axoneme) and highlight a so far unreported assocation to the flagella connector, a tiny mobile structure that connects the tip of the growing flagellum to the side of the existing flagellum.

They demonstrate that what was interpreted as basal body localisation is in fact an association to the tripartite attachment complex (TAC), a structure that links the basal body to the kinetoplast, the peculiar mass of mitochondrial DNA in these organisms. Moreover, they perform SILAC analysis with a myc-tagged version of KMP11 and unveil to which TAC protein KMP11 associates, it is the well-known TAC component termed p197. Finally, analysis of a p197 RNAi cell line at early stage of knockdown elegantly and convincingly demonstrates the specificity of the anti-KMP11 reactivity at this site: the TAC assembled before RNAi still produces signal whereas the basal body complex deprived of p197 shows drastically reduced signal. Furthermore, the actual region of p197 associating to KMP11 has been identified and validated, which is also very nice. Based on the

© 2026. Published by The Company of Biologists under the terms of the Creative Commons Attribution License (https://creativecommons.org/licenses/by/4.0/).

potential structure adopted by KMP11, the authors propose that it could cross-link different cytoskeletal molecules, hence explaining its multiple locations and its essential function.

Overall, this is a clean and well-executed study that will be of interest to the molecular and cellular parasitology community, especially since KMP11 has been proposed as drug/vaccine target. My only concern is a potential cross-reactivity of the antibody with cytoskeletal proteins containing similar domains as KMP11, but this could be addressed as suggested below.

Main concern. To demonstrate that the signal produced by the anti-KMP11 antibody is indeed specific, I would suggest reproducing the IFA experiments using the available KMP11 RNAi cell line analysing the early stage of knockdown as nicely performed by the authors for p197. This is common practise in the field and would definitely lift any ambiguity.

Other comments.
Title/abstract. The title does not really reflect the paper as it gives the impression that KMP11 was only known to be associated to the FAZ. The abstract mixes KMP11 localisation to structures (FAZ, PFR etc) and molecules (p197), this is confusing. Both titles and abstract can be improved to give better credit to the nice work reported here.

KMP11 has also been localised by the TrypTag consortium (Billington et al 2023). Although this global study does not have the level of resolution achieved here by U-ExM, it should be discussed since it partially answers the concern raised above. Similarly, the localisation of the myc-tagged version of KMP11 should be described (line 123). I assume it is similar to that reported for the endogenous protein using the antibody or to the one reported in the literature but this is not described.

More details should be provided for the production of the new antibodies. How were selected the two peptides used for immunication? What was the immunisation protocol (adjuvants etc.)?

Fig. 2. I would suggest swapping the cartoon and the IFA result. If the current order is conserved, I would then suggest adding the KMP11 localisation on the cartoon (and its reduction in KD conditions) to help visualisation for non-trypanosome people.

Minor comments

The term "intriguingly" is used 4 times, I know KMP11 is an intriguing protein, but please tone down a bit.

Ref 15-16: A reference to IFA data with anti-PFR antibodies would be more appropriate (for example Kohl et al JEM1999 or equivalent)

Lines 111-112. While it is true that the FC is specific to procyclic parasites, depletion of its components does not lead to flagellum detachment (see Varga et al PNAS2017). I would suggest deleting this statement.

Line 114. axoneme (not axonome)

Line 128. Delete "w"

Line 371. "flagella connector" (not flagellar), paraflagellar (one word)

Philippe Bastin, Institut Pasteur, Paris

---

**First revision**

<u>Author response to reviewers' comments</u>

© 2026. Published by The Company of Biologists under the terms of the Creative Commons Attribution License (https://creativecommons.org/licenses/by/4.0/). 

**Reviewer 1: SUMMARY OF THE ADVANCE MADE IN THIS PAPER AND ITS POTENTIAL SIGNIFICANCE TO THE FIELD**

In this short report, Aeschlimann et al. generated a polyclonal antibody against T. brucei KMP11, a kinetoplastid specific protein with diverse cellular distribution and essential functions, yet unclear mechanisms. Using this antibody, the authors reported KMP11 association with TAC, MtQ, HC and FC but not basal bodies. Based on previously characterized KPM11 interactions and SILAC-IP performed in this study, the authors proposed that KMP11 might bind promiscuously to cytoskeletal proteins containing alpha helices, thus explaining their widespread distribution to multiple cytoskeletal elements. A crosslinking function of KMP11 is also speculated.

SUGGESTIONS TO AUTHORS

Major comments
1. I could not find any validation of the specificity of the anti-KMP11 antibody used in this study. As several new cellular localizations of KMP11 are reported using this antibody, control for antibody specificity is essential. This antibody was used for IF, U-ExM as well as Western blots, validation of its specificity to KMP11 (e.g. loss of signal upon KMP11-RNAi, purified KMP11 protein for competition analyses etc) need to be shown for all above experimental conditions.

We now add two supplementary figures (Figs. S1, S2) to validate the specificity of the anti-KMP11 antibody.

We analysed RNAi based depletion of KMP11 by immunofluorescence microscopy, however the onset of the phenotype is so rapid and drastic affecting multiple parts of the cell that it becomes very difficult to decipher direct from indirect effects. We could however show that RNAi for 18/24 hours leads to the depletion of the major protein product of about 11 kDa (the size of KMP11) by western blot (Fig. S1). Furthermore, a large fraction of these cells displays one detached flagellum (the new flagellum, Fig. S1) supporting the involvement of KMP11 in the FAZ and potentially FC biogenesis.

Additionally, we generated cell lines inducibly expressing ectopic KMP11-myc. U-ExM analysis of these cells demonstrates that the localization pattern of KMP11-myc is consistent with that observed for endogenous, untagged KMP11 (Fig. S2). Specifically, ectopically expressed KMP11-myc localizes to the FAZ, PFR, TAC, and FC, in agreement with the localization pattern detected using the anti-KMP11 antibody.

2. The proposed model that KMP11 interacts promiscuously with alpha helixes is interesting, but lacks strong experimental support. The interaction between KMP11 and p197 central helical region is shown by co-IP. The model would be stronger if supported by direct binding using purified components. The cross- species result (binding to T. cruzi p197 central domain) is interesting, but it still doesn't exclude the presence of "bridging proteins" conserved across trypanosomes.

We thank the reviewer for these thoughtful comments and fully agree that our proposed model currently remains speculative and that is why we explicitly frame it as a working hypothesis rather than a demonstrated mechanism.

As discussed in the manuscript, the observation that KMP11 localizes to such a diverse set of cytoskeletal structures is highly unusual. A common feature of these structures is their enrichment in proteins containing extensive α-helical and coiled-coil domains. This led us to speculate that KMP11 may recognize a shared structural feature rather than a defined amino acid sequence. In support of this idea, we show that *T. brucei* KMP11 interacts not only with the central domain of *T. brucei* p197, but also with the corresponding central domain of *T. cruzi* p197, despite the absence of detectable sequence homology between the two proteins. Importantly, both domains are predicted to be highly α-helical and coiled-coil rich. Furthermore, our SILAC-IP analysis reveals that proteins enriched more than fivefold in KMP11 pull-downs display a significantly higher average α-helical content than less enriched proteins. Together, these observations are consistent with, although clearly not proof of, the possibility that KMP11 preferentially associates with α-helical/coiled-coil structures. Furthermore, we fully acknowledge the reviewer's point that the

© 2026. Published by The Company of Biologists under the terms of the Creative Commons Attribution License (https://creativecommons.org/licenses/by/4.0/).

current co-IP experiments cannot exclude the involvement of conserved bridging proteins. Demonstrating direct interaction using purified components would indeed considerably strengthen the model. However, establishing such biochemical assays for these very large and highly coiled-coil-rich proteins is technically challenging and unfortunately beyond the scope of the present short report. We believe that our current data is sufficient for a short report. And we are of course following up on these questions in future work.

3. Even for a short report, I think the speculated crosslinking function of KMP11 requires some test. Specifically, whether KMP11 depletion affects the organization/spacing/filament properties of p197 or other cytoskeletal proteins, in vivo or in vitro?

Similarly, we agree that the proposed crosslinking function of KMP11 is currently hypothetical. In the manuscript, we only intended to suggest a conceptual framework that could potentially explain the diverse localization pattern of KMP11. The idea is partly motivated by the known antiparallel homodimeric structure of KMP11, which is reminiscent of the overall quaternary organization of α- actinin, a well-characterized actin crosslinker. Nevertheless, we fully agree that direct experimental evidence demonstrating effects of KMP11 depletion on p197 filament organization, spacing, or cytoskeletal architecture is currently lacking. Addressing these questions will require substantial future work, including structural and biochemical analyses, which are beyond the scope of the current study (a short report).

4. KMP11 was first reported in Leishmania as a membrane protein. To my knowledge, its membrane association is not tested in T. brucei, even though purified KMP11 forms structured 4-helix bundle in DPC micelles. While the proposed model and discussion focused on KMP11 binding to various cytoskeletal elements, its membrane association, even possible membrane-cytoskeleton crosslinking shall not be overlooked.

Our current imaging data does not indicate any membrane association, and in a preliminary set of experiments we have verified the insolubility of the majority of KMP11 by digitonin fractionation. Thus, at least the majority seems not to be membrane bound (see figure below). Since we are already at the maximum of figures for a short report and have already added two additional supplementary figures we would prefer not to add this dataset. To comply with the request of the reviewer we have changed a sentence in the conclusions from: "*These localizations imply that trypanosomal KMP11 does not need to directly interact with lipid bilayers, unlike leishmanial KMP11" to: " These localizations suggest that the majority of trypanosomal KMP11 does not need to directly interact with lipid bilayers, unlike the leishmanial KMP1. However, we cannot exclude that a small part of KMP11 might associate also with lipids*".

NOTE: We have removed unpublished data that had been provided for the referees in confidence.

**Figure for reviewer Majority of KMP11 remains insoluble in 1% digitonin**. Comparison of different biochemical fractions via Western Blot. Whole cells served as input for the first fractionation with 0.015% digitonin. The Pellet of the first fractionation served as input for the second fractionation with 1% digitonin. The membrane was stained for KMP11, VDAC and EF1a. Molecular weight markers (kDa) are indicated on the left.

Minor comments

The SILAC-IP study was performed on cells with tetracycline-induced KMP11-myc expression (from pLEW100). Characterization of this cell line is not shown. Importantly, how much is KMP11-myc overexpression compared to native KMP11? Does overexpressed KMP11-myc show the same cellular localizations as native KMP11? These are important, considering the potentially promiscuous nature of KMP11 binding.

We have now added two supplementary figures to address these questions. Fig. S2 shows the localization of the tetracycline-induced KMP11-myc expression to be like the one shown for the native protein by antibody staining (Fig. 1). As for the expression levels we have added Fig. S3 that shows, that in a Western blot analysis the protein levels of ectopically expressed KMP11-myc were substantially lower than those of endogenous KMP11, rendering the ectopically expressed

© 2026. Published by The Company of Biologists under the terms of the Creative Commons Attribution License (https://creativecommons.org/licenses/by/4.0/).

KMP11-myc undetectable with the KMP11 antibody (Fig. S3A, B).

For both KMP11-myc and native KMP11, how soluble are these proteins? The cells were solubilized with 1% digitonin in the SILAC-IP experiments. How much KMP11 remains insoluble under this condition? Focusing on the soluble fraction for coIP in this study is OK, but I am afraid most (and some of the strongest) KMP interactions may be overlooked in the insoluble fractions.

We agree with the reviewer. In our experience with cytoskeletal proteins such as the TAC proteins only a very small portion becomes/is soluble (see above). However, we are also always able to detect interacting proteins likely because they pre-assemble in pre-complexes prior to them being integrated in insoluble structures. We have added a sentence to the results section to clarify this: “It is worth noting that the majority of KMP11 remains insoluble after lysis with detergents such as digitonin, suggesting that very strong interactors might have been missed in the proteomics analysis”.

**Reviewer 2: MS ID#: jcs.264898**
**MS Title: From the FAZ to the TAC: Widespread Cytoskeletal Engagement**

Authors: Aeschlimann et al

This interesting manuscript revisits the localisation of KMP11, a unique Kinetoplastid protein which has puzzled the community since its discovery because of its localisation to multiple structures. The authors generated a new antibody (targeting a couple of KMP11 peptides) and using Ultrastructure Expansion Microscopy (U-ExM), they refine KMP11 location at the flagellum level (paraflagellar rod - PFR - rather than axoneme) and highlight a so far unreported assocation to the flagella connector, a tiny mobile structure that connects the tip of the growing flagellum to the side of the existing flagellum.
They demonstrate that what was interpreted as basal body localisation is in fact an association to the tripartite attachment complex (TAC), a structure that links the basal body to the kinetoplast, the peculiar mass of mitochondrial DNA in these organisms. Moreover, they perform SILAC analysis with a myc- tagged version of KMP11 and unveil to which TAC protein KMP11 associates, it is the well-known TAC component termed p197. Finally, analysis of a p197 RNAi cell line at early stage of knockdown elegantly and convincingly demonstrates the specificity of the anti-KMP11 reactivity at this site: the TAC assembled before RNAi still produces signal whereas the basal body complex deprived of p197 shows drastically reduced signal. Furthermore, the actual region of p197 associating to KMP11 has been identified and validated, which is also very nice. Based on the potential structure adopted by KMP11, the authors propose that it could cross-link different cytoskeletal molecules, hence explaining its multiple locations and its essential function.

Overall, this is a clean and well-executed study that will be of interest to the molecular and cellular parasitology community, especially since KMP11 has been proposed as drug/vaccine target. My only concern is a potential cross-reactivity of the antibody with cytoskeletal proteins containing similar domains as KMP11, but this could be addressed as suggested below.

We would like to thank the reviewer for the thoughtful comments that we address below.

Main concern. To demonstrate that the signal produced by the anti-KMP11 antibody is indeed specific, I would suggest reproducing the IFA experiments using the available KMP11 RNAi cell line analysing the early stage of knockdown as nicely performed by the authors for p197. This is common practise in the field and would definitely lift any ambiguity.

We agree with the reviewer and now add two supplementary figures (S1, S2) to validate the specificity of the anti-KMP11 antibody.

We analysed RNAi based depletion of KMP11 by immunofluorescence microscopy; however the phenotype develops so rapidly affecting multiple parts of the cell that it becomes very difficult to decipher direct from indirect effects. We could however show that RNAi for 18/24 hours leads to the depletion of the major protein product on of about 11 kDa by western blot (KMP11 is 11 kDa, Figure S1).

© 2026. Published by The Company of Biologists under the terms of the Creative Commons Attribution License (https://creativecommons.org/licenses/by/4.0/). 

Furthermore, a large fraction of these cells displays one detached flagellum (the new flagellum, Fig. S1) supporting the involvement in the FAZ and potentially FC biogenesis.

Additionally, we generated cell lines inducibly expressing ectopic KMP11-myc. U-ExM analysis of these cells demonstrates that the localization pattern of KMP11-myc is consistent with that observed for endogenous, untagged KMP11 (Fig. S2). Specifically, ectopically expressed KMP11-myc localizes to the FAZ, PFR, TAC, and FC, in agreement with the localization pattern detected using the anti-KMP11 antibody.

Other comments.

Title/abstract. The title does not really reflect the paper as it gives the impression that KMP11 was only known to be associated to the FAZ. The abstract mixes KMP11 localisation to structures (FAZ, PFR etc) and molecules (p197), this is confusing. Both titles and abstract can be improved to give better credit to the nice work reported here.

We would rather think that the title captures the broad cytoskeletal interaction network of KMP11 and would prefer to keep the title as is.

We changed the abstract to: KMP11 is a conserved kinetoplastid protein yet its precise localization and function remained unclear. Using immunofluorescence, ultrastructure expansion microscopy (U-ExM), and proteomics, we comprehensively analyzed KMP11 in procyclic *Trypanosoma brucei*. Besides confirming localization to the flagellum attachment zone (FAZ), we identify previously unknown localizations at the paraflagellar rod (PFR), flagellar connector (FC), microtubule quartet (MtQ), and flagellar pocket collar/hook complex (FPC/HC) region. We further show that the previously described basal body localization corresponds to the tripartite attachment complex (TAC). Proteomic analyses identify multiple cytoskeletal interactors, including the TAC component p197. Immunoprecipitation and U-ExM analyses demonstrate that KMP11 associates with the central, alpha helical domain of p197 from both *T. brucei* and *T. cruzi*. Furthermore, KMP11 interactors overall display significantly elevated α- helical content. Together, our findings establish KMP11 as a component of a broad cytoskeletal interaction network in trypanosomes and we speculate that KMP11 preferentially associates with α- helical proteins.

KMP11 has also been localised by the TrypTag consortium (Billington et al 2023). Although this global study does not have the level of resolution achieved here by U-ExM, it should be discussed since it partially answers the concern raised above. Similarly, the localisation of the myc-tagged version of KMP11 should be described (line 123). I assume it is similar to that reported for the endogenous protein using the antibody or to the one reported in the literature but this is not described.

We added the reference to the TrypTag consortium. For the localization of the myc-tagged version please see above.

More details should be provided for the production of the new antibodies. How were selected the two peptides used for immunication? What was the immunisation protocol (adjuvants etc.)?

We elaborated on the antibody production process in the materials and methods section: Polyclonal rabbit antiserum against TbKMP11 was commercially produced (Eurogentec, Belgium, 87 days -Immunization of the 2 animals with the mix of coupled peptides) using aa 26-40 (QNKRFFADKPDEATL), and aa 78-92 (AELLEQQKNAQFPGK) as antigens. The Peptides were suggested by the company and according to AlphaFold prediction, they localize in the helix kink and at the very C-terminus. For both peptides, 5 ml of serum was purified against the peptide.

Fig. 2. I would suggest swapping the cartoon and the IFA result. If the current order is conserved, I would then suggest adding the KMP11 localisation on the cartoon (and its reduction in KD conditions) to help visualisation for non-trypanosome people.

© 2026. Published by The Company of Biologists under the terms of the Creative Commons Attribution License (https://creativecommons.org/licenses/by/4.0/).

We changed the figure accordingly.

Minor comments

The term "intriguingly" is used 4 times, I know KMP11 is an intriguing protein, but please tone down a bit.

We agree and change the sentences accordingly.

Ref 15-16: A reference to IFA data with anti-PFR antibodies would be more appropriate (for example Kohl et al JEM1999 or equivalent)

We added the reference.

Lines 111-112. While it is true that the FC is specific to procyclic parasites, depletion of its components does not lead to flagellum detachment (see Varga et al PNAS2017). I would suggest deleting this statement.

We removed the statement, although it remains peculiar as to why depletion of KMP11 would affect PCF but not the BSF flagella attachment if it is not related to the FC.

Line 114. axoneme (not axonome)

We corrected axoneme

Line 128. Delete "w"

We deleted the extra w

Line 371. "flagella connector" (not flagellar), paraflagellar (one word)

We corrected the two spelling mistakes

Philippe Bastin, Institut Pasteur, Paris

---

Second decision letter

MS ID#: jcs.264898R1

MS Title: From the FAZ to the TAC: Widespread Cytoskeletal Engagement of T. brucei KMP11

Authors: Salome Aeschlimann; Clirim Jetishi; Caroline E. Dewar; Philip Stettler; Markus Gerber; Silke Oljeklaus; Bettina Warscheid; Torsten Ochsenreiter; AndrÃ© Schneider

Article Type: Short Report

Dear Dr Ochsenreiter,

I am happy to tell you that your manuscript has been accepted for publication in Journal of Cell Science, pending standard publication integrity checks.

Reviewer 2

The authors have addressed adequately the vast majority of my comments. I am a bit surprised by the absence of anti-KMP11 staining in the RNAi cell line after a short induction time but since the

© 2026. Published by The Company of Biologists under the terms of the Creative Commons Attribution License (https://creativecommons.org/licenses/by/4.0/).

tagged version shows the same distribution, this lifts my concern of potential cross-reactivity of the antibody with other coiled-coil proteins.

Philippe Bastin, Institut Pasteur, Paris

© 2026. Published by The Company of Biologists under the terms of the Creative Commons Attribution License (https://creativecommons.org/licenses/by/4.0/).
